# Evaluating the effects of simulation training on stroke thrombolysis: a systematic review and meta-analysis

**DOI:** 10.1186/s41077-024-00283-6

**Published:** 2024-02-29

**Authors:** Sameera Aljuwaiser, Abdel Rahman Abdel-Fattah, Craig Brown, Leia Kane, Jamie Cooper, Alyaa Mostafa

**Affiliations:** 1 Cambridge University, Cambridge, UK; 2 Emergency Medicine, NHS Grampian, Aberdeen, Scotland; 3 School of Medicine, Medical Sciences and Nutrition, University of Aberdeen, Aberdeen, AB25 2ZD UK

**Keywords:** Simulation training, Door-to-needle time, Ischaemic stroke, Review

## Abstract

**Background:**

Ischaemic strokes are medical emergencies, and reperfusion treatment, most commonly intravenous thrombolysis, is time-critical. Thrombolysis administration relies on well-organised pathways of care with highly skilled and efficient clinicians. Simulation training is a widespread teaching modality, but results from studies on the impact of this intervention have yet to be synthesised. This systematic review and meta-analysis aimed to synthesise the evidence and provide a recommendation regarding the effects of simulation training for healthcare professionals on door-to-needle time in the emergency thrombolysis of patients with ischaemic stroke.

**Methods:**

Seven electronic databases were systematically searched (last updated 12th July 2023) for eligible full-text articles and conference abstracts. Results were screened for relevance by two independent reviewers. The primary outcome was door-to-needle time for recombinant tissue plasminogen activator administration in emergency patients with ischaemic stroke. The secondary outcomes were learner-centred, improvements in knowledge and communication, self-perceived usefulness of training, and feeling ‘safe’ in thrombolysis-related decision-making. Data were extracted, risk of study bias assessed, and analysis was performed using RevMan™ software (Web version 5.6.0, The Cochrane Collaboration). The quality of the evidence was assessed using the Medical Education Research Study Quality Instrument.

**Results:**

Eleven studies were included in the meta-analysis and nineteen in the qualitative synthesis (*n* = 20,189 total patients). There were statistically significant effects of simulation training in reducing door-to-needle time; mean difference of 15 min [95% confidence intervals (CI) 8 to 21 min]; in improving healthcare professionals’ acute stroke care knowledge; risk ratio (RR) 0.42 (95% CI 0.30 to 0.60); and in feeling ‘safe’ in thrombolysis-related decision-making; RR 0.46 (95% CI 0.36 to 0.59). Furthermore, simulation training improved healthcare professionals' communication and was self-perceived as useful training.

**Conclusion:**

This meta-analysis showed that simulation training improves door-to-needle times for the delivery of thrombolysis in ischaemic stroke. However, results should be interpreted with caution due to the heterogeneity of the included studies.

**Supplementary Information:**

The online version contains supplementary material available at 10.1186/s41077-024-00283-6.

## Background

Stroke is the second-leading cause of mortality worldwide [1]. The vast majority of strokes have an ischaemic pathogenesis [2–4] though underlying mechanisms may be variable and complex [3]. From the onset of clinical symptoms, the ischaemic core is surrounded by neurons that may remain viable for several hours prior to the development of irreversible ischaemic injury [5, 6]. This affords a treatment window where prompt restoration of blood supply may permit the survival of the threatened neurons, known as the penumbra [6–9]. The determinants of whether cerebral ischaemia leads to infarction are anatomical (relating to the presence and extent of protective collateral circulation) and time-critical, with respect to having access to reperfusion treatment [10, 11]. Stroke patients identified early have the greatest potential to benefit from reperfusion either via mechanical thrombectomy (currently restricted mainly to specialised centres) [12] but more commonly with intravenous thrombolysis using recombinant tissue plasminogen activator (rtPA), through therapeutic benefit diminishes with time [6–9].

Consequently, emphasis on awareness of stroke symptoms and the time-critical nature of assessment has increased through organisations like Brain Attack Coalition [13] and public health campaigns. The adoption of tools such as Facial drooping, Arm weakness, Speech difficulties, and Time (FAST), now used widely by paramedics, improves recognition and enables pre-alert of the receiving hospital to patient arrival [14]. Reduced time from the hospital door to rtPA administration (door-to-needle time) [15] alone decreases mortality and haemorrhagic transformation associated with ischaemic stroke [6, 16], with a target of under 60 min set internationally [17–19]. Therefore, clinical pathways for emergency stroke patients have to be responsive and efficient throughout, from first notification by ambulance services to the Emergency Department (ED), patient reception, computed tomography (CT) imaging, through to obtaining specialist radiology and clinical assessment to determine the best course of action, with the aim of swiftly initiating intravenous thrombolysis if appropriate [20]. Barriers to administering rtPA to those patients who may benefit include clinician uncertainty regarding the administration of treatment with the potential to cause harm and lack of practice in delivery [21, 22]. Current stroke guidelines urge the establishment of educational initiatives to improve outcomes in patients presenting as emergencies with ischaemic stroke [23].

Simulation training has been widely used as an educational modality in several specialties with “time-dependent” processes such as trauma care and life support [24–26]. However, adoption has been slower within the neurological sciences [27], and evidence suggests that human factors are the most significant rate-limiting component in the delivery of emergency care to stroke patients [28]. In this clinical context, simulation training may provide an opportunity for teams to increase knowledge and develop the processes, skills, and teamwork required to optimise the safe delivery of intravenous thrombolysis in educationally beneficial representations of real-world environments [12, 29].

The effectiveness of simulation training on the investigated outcomes can be assessed using Kirkpatrick’s Four-Level Training Evaluation model, which identifies the effects of particular training on the organisation level and patients as a whole [30], and is considered the reference standard for evaluation of training in healthcare contexts [31].

Although there are numerous primary studies on the effects of simulation training on door-to-needle time, to the authors’ knowledge, no meta-analysis on this topic exists. This systematic review and meta-analysis aimed to address the gap in the literature by assessing the effects of simulation training for healthcare professionals on door-to-needle time delivery of emergency thrombolysis in ischaemic stroke.

## Methods

### Study design

This systematic review and meta-analysis were performed per the guidelines of the Cochrane Handbook of Systematic Reviews of Interventions [32] and the Preferred Reporting Items for Systematic Review and Meta-Analysis (PRISMA) statement [33]. Appendix 1 details the PRISMA checklist. The objective was to synthesise the available evidence regarding the effects of simulation training for healthcare professionals on door-to-needle time delivery of emergency thrombolysis in ischaemic stroke patients.

### Study eligibility

Any study investigating healthcare professional simulation training with respect to intravenous thrombolysis administration in stroke patients versus no intervention was eligible for inclusion, with the primary outcome being door-to-needle time and learner-centred secondary outcomes. Simulation training or activity was defined as the complete set of events and actions that occur from initiation to termination of a particular simulation event [34]. No intervention was defined as any period without simulation training.

Table 1 illustrates the eligibility criteria.

**Table 1:** Inclusion and exclusion criteria using the Participants, Intervention, Comparisons, and Outcomes (PICO) Framework [32]

	Inclusion criteria	Exclusion criteria
Study design	All study types and conference abstracts	Books, Commentaries, Editorials, Guidelines, Letters, News and Opinions, Reports and Reviews
Participants	All qualified (postgraduate) healthcare professionals in clinical practice or clinical training who are involved with intravenous thrombolysis administration as a treatment for ischaemic stroke	Healthcare (undergraduate) students or professionals in training Healthcare professionals not involved with intravenous thrombolysis in the management of ischemic stroke
Intervention	Any form of simulation training for ischaemic stroke intravenous thrombolysis administration	Other forms of teaching interventions. Training on other treatments for stroke that are not intravenous thrombolysis
Comparisons	No interventions/no simulation training (e.g. continued postgraduate training without any forms of simulation, no change to training curriculums)	
Outcomes	The primary outcome of door-to-needle time for intravenous thrombolysis administration The learner-centred secondary outcomes of improvement in stroke knowledge and/or feeling ‘safe’ in thrombolysis-related decision-making and/or self-perceived usefulness of simulation training and/or improvement in communication	Other outcomes

### Study identification

The literature search was first conducted on 17th May 2023 and last updated on 12th July 2023 using EMBASE, PubMed, PsycINFO, ERIC, CINAHL, Scopus, and Google Scholar. The entry date was 1990 when the results of the first recombinant tissue plasminogen activator (rtPA) trial were published, which was followed by the United States Food and Drug Administration’s approval for rtPA as a treatment for acute ischaemic stroke in 1996 [35].

The search was performed by two independent researchers (SA and AA). The search strategy included MeSH and text search terms, agreed upon by the research team, combined with Boolean operators “AND” and “OR”. Details of the search strategies are listed in Appendix 2.

Electronic search strategies were limited to adult humans (over 18 years old), and no restrictions on language or publication types were applied. Citation lists of included publications were manually scrutinised for additional relevant studies, and a manual search of international conference abstract databases was performed, including the Association for Simulated Practice in Healthcare [36], Society for Simulation in Europe [37], International Meeting on Simulation in Healthcare [38], Australasian Simulation Congress [39], and the International Clinical Skills Conference [40].

### Study selection

All titles and abstracts retrieved were independently screened for relevance. Using Microsoft Excel®, duplicates were manually removed, and non-relevant articles were excluded. The full texts of all identified studies were retrieved and assessed for eligibility independently by two researchers (SA and AA). Studies meeting the eligibility criteria were included following the Cochrane Handbook for Systematic Reviews of Interventions. Discrepancies between SA and AA were discussed and agreed upon with a senior reviewer (AM), ensuring no potentially relevant papers were discarded.^32^

### Data extraction

Data were extracted based on the guidelines for health care simulation research [41] and inputted into a Microsoft Excel® spreadsheet (Appendix 3). Non-English articles were translated completely. All corresponding authors of included studies with any missing data were contacted.

### Data synthesis

Quantitative analysis was performed using RevMan™ (Web version 5.6.0, The Cochrane Collaboration) [42]. A meta-analysis was performed for the primary outcome, with results expressed as mean difference with 95% confidence intervals (CI) and secondary outcome results expressed as risk ratio (RR) with 95% CI. Where possible, median and interquartile range (IQR) values were converted to mean $\left(\overline{{\text{x}} }\right)$, and standard deviation (SD) using the $\overline{{\text{x}} }=\frac{{Q}_{1}+median+{Q}_{3}}{3}$ and ${\text{SD}}=\frac{{Q}_{3}-{Q}_{1}}{1.35}$ formulae [43] (Q_1_ = 25th and Q_3_ = 75th percentiles). A *p*-value of < 0.05 was considered statistically significant. Heterogeneity between studies was assessed by the *I*^*2*^ score, using the random-effects meta-analysis model to account for data heterogeneity [32]. Two sensitivity analyses were performed for the primary outcome. The first included studies eligible for meta-analysis with low-to-moderate risk of bias and the second, studies of moderate-to-high methodological quality. Subgroup analysis was not performed for the primary outcome due to insufficient details on patient characteristics being available.

### Assessment of risk of bias and study quality

The Cochrane ‘Risk of Bias in Non-Randomised Studies of Interventions’ (ROBINS-I) tool was used to assess the risk of bias [44], with graphs generated by Robvis [45]. Appendix 4 includes details of the ROBINS-I assessment. The quality of the included studies was assessed using the Medical Education Research Study Quality Instrument (MERSQI); low-quality studies scored MERSQI ≤ 12 [46].

## Results

### Identification of studies and study selection

The complete search strategy identified 1590 potentially relevant articles. After duplicate removal and independent assessment of titles and abstracts for relevance, 287 were selected for full-text review. Nineteen studies [47–65] met the inclusion criteria and were included in the systematic review, ten full-text articles [47, 51, 52, 55–58, 60, 63, 65] and nine conference abstracts [48–50, 53, 54, 59, 61, 62, 64]. Eleven studies with complete data were included in the meta-analysis [47, 49, 51, 52, 55–58, 60, 63, 65].

**Fig. 1:**
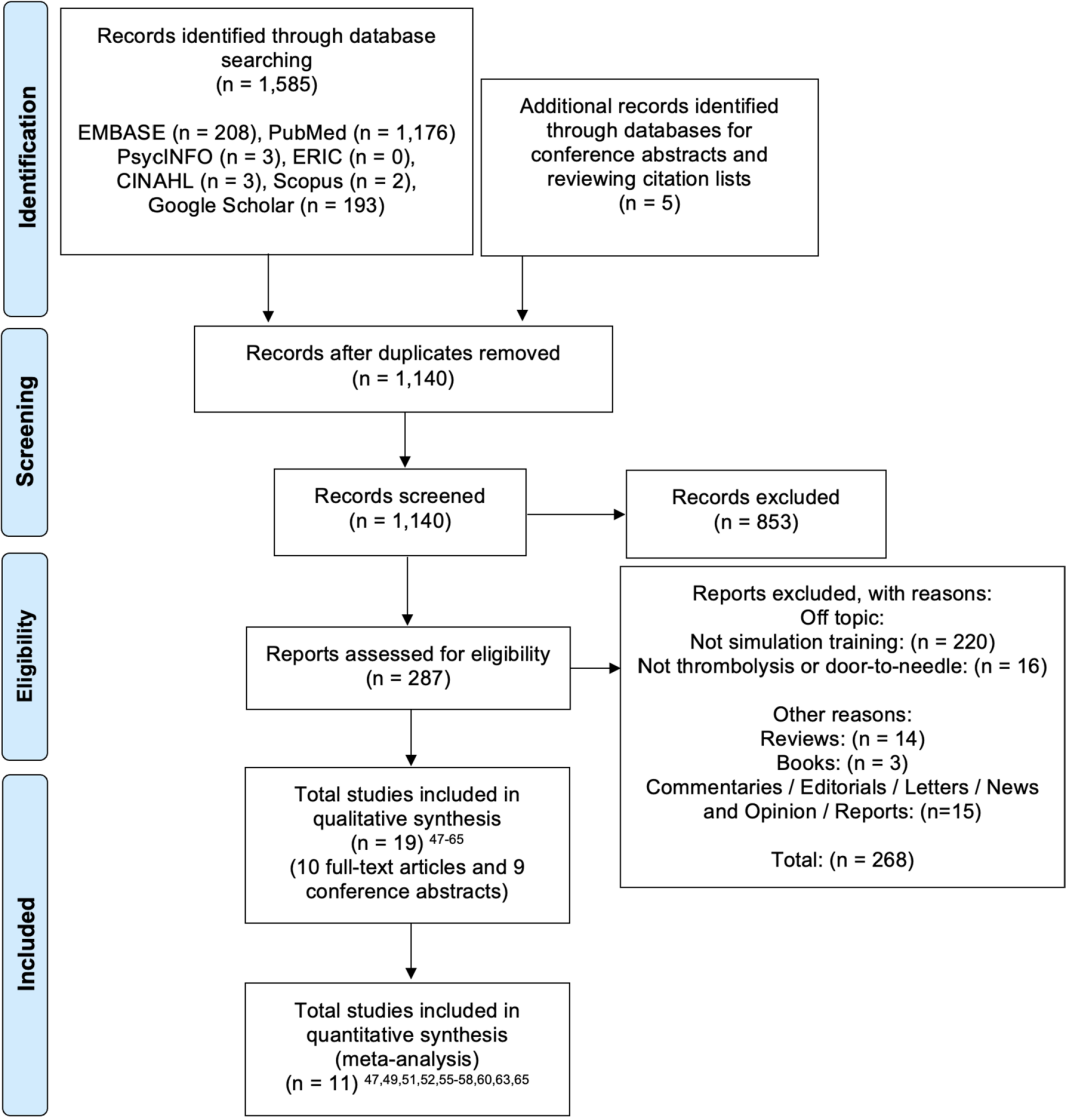
Flowchart according to Preferred Reporting Items for Systematic Reviews and Meta-Analysis (PRISMA) methodology

Figure 1 outlines the PRISMA flow diagram following the Cochrane Handbook for Systematic Reviews of Interventions [32].

### Study characteristics

Of the nineteen included studies published between 2016 and 2023, seven were conducted in the USA [50, 51, 53, 55, 58, 61, 64] three in Germany [47, 52, 63], two in Australia [54, 59] and one each in Austria [62], Brazil [56], Czech Republic [65], France [57], Japan [49], Norway [60], and the United Kingdom (UK) [48]. One full-text article published in German was translated [47]. Eleven studies included a total number of 20,189 patients [47, 49, 51, 52, 55–58, 60, 63, 65], and the remaining eight studies did not report patient numbers [48, 50, 53, 54, 59, 61, 62, 64]. Thirteen studies included 1197 healthcare professionals [47, 50–53, 55–58, 60, 61, 63, 65]; and the remaining six did not report participant numbers.^48,49,54,59,62,64^ All studies compared door-to-needle times before and after simulation training. Thirteen studies reported multidisciplinary cooperation [47–49, 52–54, 56, 57, 59, 60, 62, 63, 65], five focused on physicians [50, 51, 55, 58, 61], and one on nurses [64]. Manikins were used for simulation training in five studies [47, 50, 52, 56, 63], four studies used hospital staff as patients [53, 57, 58, 65] four used simulated patients [55, 59–61], (one specifically recruiting previous stroke patients) [60], and six did not specify [48, 49, 51, 54, 62, 64].

Three studies utilised Crew Resource Management aspects in reducing door-to-needle times [47, 52, 63]. Crew Resource Management is a training concept focusing on non-technical and behavioural skills such as situational awareness, decision-making, leadership, and teamwork [66, 67].

Four studies evaluated improvements in knowledge [47, 51, 52, 55], and four assessed the self-perceived usefulness of simulation training [52, 60, 63, 65]. Two studies assessed feeling ‘safe’ in thrombolysis-related decision-making [47, 52], and two evaluated improvements in communication [47, 65]. Concurrent with the introduction of simulation training, five studies underwent stroke protocol revisions [49, 54, 55, 60, 62] three used multifaceted interventions [47, 48, 54] and one began another door-to-needle time-related project in their Emergency Department (ED) 6 months after the introduction of the simulation intervention [51].

Table 2 presents the characteristics of the included studies**. **Appendix 5 presents a list of excluded studies (*n* = 32 studies).

**Table 2:** Characteristics of included studies

Author; year, country	Participants, setting,	Intervention (type of simulation training)	Length of training	Outcome measure	MERSQI
Tahtali et al. 2016 [47] Germany	Number of patients: • Pre-simulation training: 50 patients • Post-simulation training: 83 patients Number of learners: 151 Healthcare professionals: 8 neurology specialists, 39 neurology residents, 8 neuroradiology residents, 28 emergency department nurses, 5 neuroradiology and medical-technical radiology assistants, 57 students, and 6 external guests Hospital: Neurological Emergency Room at the Centre for Neurology and Neurosurgery	The simulation training includes a remote-controlled manikins, connected to a real clinic monitor, that is used for the simulated scenarios with variable circulatory parameters throughout the simulation. The stroke team simulation was based on the concepts of Crew Resource Management	Monthly training in small groups with up to eight participants, consisting of new stroke team members, for 2 years	Door-to-needle time^a^ Improvement in intravenous thrombolysis knowledge Feeling safe making decisions in acute stroke care Improvement in communication	12.0
Waterson et al. 2016 [48] United Kingdom	Number of patients: not specified Number of learners: not specified Healthcare professionals: medical and surgical staff Hospital: emergency department	In-situ simulation training (simulated scenarios)	One-off simulation training session	Door-to-needle time	11.0
Ohara et al. 2017 [49] Japan	Number of patients: • Pre-simulation training: 46 patients • Post-simulation training: 36 patients Number of learners: not specified Healthcare professionals: acute stroke team (multidisciplinary team) Hospital: (single centre)—not specified	Simulation training by organising in-hospital lectures and simulation training courses	Not specified	Door-to-needle time	11.5
Richardson et al. 2017 [50] USA	Number of patients: not specified Number of learners: 4 Healthcare professionals: Neurology residents (first years—PGY-1) Hospital: simulation-based learning environment	Simulation-based learning scenarios were developed, using a manikin controlled by the simulation lab personnel. Participants were videotaped performing the scenario, which was incorporated into their debrief	One-off simulation training session	Door-to-needle time	12.0
Ruff et al. 2017 [51] USA	Number of patients: • Pre-simulation training: 72 patients • Post-simulation training: 98 patients Number of learners: 15 Healthcare professionals: Neurology Residents (PGY-2, PGY-3, and PGY-4) Hospital: emergency department	Case-based simulation course with Socratic presentation of acute stroke cases (based on 4 scenarios). The boot camp was facilitated by senior residents, stroke fellows, and stroke attending physicians	One-off resident bootcamp	Door-to-needle time^a^ Improvement in intravenous thrombolysis knowledge	11.5
Tahtali et al. 2017 [52] Germany	Number of patients: • Pre-simulation training: 122 patients • Post-simulation training: 112 patients Number of learners: 176 Healthcare professionals: physicians, nurses, and technicians) participated in on-site stroke team simulation training from seven hospitals. 152 healthcare professionals completed the questionnaires from 6 stroke units (University Hospital Frankfurt did not participate) Hospitals: (Interdisciplinary Neurovascular Network)—three comprehensive and four regional stroke units (total of seven stroke units)	Train-the-trainer seminar to educate stroke team trainers for each stroke unit conveying the principles of Crew Resource Management Simulation-based training with the simulation team in each participating hospital using a high-fidelity manikin. The remote-controlled high-fidelity manikin, connected to a lifelike monitor, was filled with artificial blood, and placed on a stretcher, mimicking stroke-like symptoms	Two and a half hours of stroke team training over 2 months throughout the seven participating hospitals	Door-to-needle time^a^ Improvement in intravenous thrombolysis knowledge Self-perceived usefulness of simulation training Feeling safe making decisions in acute stroke care	13.0
Tse-Chang et al. 2017 [53] USA	Number of patients: not specified Number of learners: 187 Healthcare professionals: 153 emergency department nurses, 8 emergency department physicians, 6 neurologists, 4 pharmacists, 6 radiology technicians, and 10 phlebotomists Hospital: Emergency Department	The simulation training included code stroke responders (five nurses) and the scenarios demonstrated right hemispheric syndrome. Participants in the simulation took a focused history and relayed their findings to the neurologist to evaluate the inclusion/exclusion criteria and the administration of thrombolysis	One-off simulation training session with a 90-min scenario	Door-to-needle time	12.0
Windle et al. 2017 [54] Australia	Number of patients: not specified Number of learners: not specified Healthcare professionals: Stroke team, emergency, and clinical education clinicians, in addition to radiology, anaesthetics, administration and communications staff Hospital: tertiary centre. Off-site (hospital simulation centre) and in-situ simulation (emergency department)	Phase I of the simulation training included patient scenario for the stroke team Phase II was the in-situ simulation in the emergency department; video footage was used to guide improvement	Four off-site simulations and one in-situ simulation for stroke education	Door-to-needle time	10.5
Zidan et al. 2017 [55] USA	Number of patients: • Pre-simulation training: 34 patients • Post-simulation training: 41 patients Number of learners: 13 Healthcare professionals: Resident physicians (7 PGY-2 and 6 PGY-3) Hospital: Simulation Lab	Mock cases (clinical scenarios) of code stroke, were created, replicating real-life events on standardised patients. The cases included clinical vignettes	Thirteen stroke cases over one day (the cases included history of symptoms, lab data, and radiological images)	Door-to-needle time^a^ Improvement in intravenous thrombolysis knowledge	12.0
Carvalho et al. 2018 [56] Brazil	Number of patients: • Pre-simulation training: 90 patients • Post-simulation training: 199 patients Number of learners: 122 Healthcare professionals: from stroke facilities and pre-hospital care Hospital: three emergency clinics and two hospitals	Case vignettes of a fictitious scenario, involving healthcare professionals as members of the team/patients family with challenges such as anxious family members and emergency department staff making wrong decisions. The simulated cases are based on a manikin (ALS Simulator, Laerdal Medical) able to mimic blood pressure, heart sounds, peripheral pulse, ECG, simulates the patient on a hospital bed	Eighteen training sessions over 11 months	Door-to-needle time^a^	13.0
Haesebaert et al. 2018 [57] France	Number of patients: • Pre-simulation training: 328 patients • Post-simulation training: 363 patients Number of learners: 72 Healthcare professionals: emergency physicians and nurses Hospital: 18 emergency units (simulated environments)	Interactive simulation using clinical cases played by two stroke unit nurses to identify the Face Arm Speech Time tool for stroke detection Simulation training by physicians to perform the National Institutes of Health Stroke Scale score after watching the French national neurovascular society video on simulated patients	One day of training Aimed to improve the knowledge and skills of triage nurses in detecting strokes and using the National Institutes of Health Stroke Scale score by emergency physicians	Door-to-needle time^a^	14.5
Mehta et al. 2018 [58] USA	Number of patients: • Pre-simulation training: 172 patients • Post-simulation training: 276 patients Number of learners: 20 Healthcare professionals: PGY-2 neurology residents Hospital: neurology department	Mock code stroke simulations used trained live actors (neurology nurses), portraying stroke vignettes and depicting focal neurological findings correlating with each case	One session over one day, every year for current PGY-2 neurology residents	Door-to-needle time^a^	13.5
Sanders et al. 2018 [59] Australia	Number of patients: not specified Number of learners: not specified Healthcare professionals: stroke team Hospital: not specified	Simulation training was based on a real case of right middle cerebral artery occlusion and was adapted depending on the skill set of each participating group. The scenarios included simulated patients	One session every rotation over 1 year	Door-to-needle time	11.0
Ajmi et al. 2019 [60] Norway	Number of patients: • Pre-simulation training: 399 patients • Post-simulation training: 190 patients Number of learners: 210 Healthcare professionals: stroke physicians, radiologists, paramedics, interventional radiologists, emergency room nurses, neuroradiologists, radiographers, and neurology registrars Hospital: stroke units and emergency rooms	Previous stroke patients acted as simulated patients for the in-situ simulation-based training sessions that included scripted scenarios, mimicking real-life cases	One weekly session (lasting approximately 60 min) with a 4-month pause. Total of 20 simulation sessions	Door-to-needle time^a^ Self-perceived usefulness of simulation training	13.0
Singh et al. 2019 [61] USA	Number of patients: not specified Number of learners: 24 healthcare professionals: internal medicine residents Hospital: community hospital	Acute stroke simulation included two different case scenarios using standardised patients (one acute ischaemic stroke case within 3 h and an acute ischaemic stroke within 24 h). The simulation session was videotaped	One-off session for all internal medicine residents at a community hospital before starting stroke calls	Door-to-needle time	9.0
Bubel et al. 2020 [62] Austria	Number of patients: not specified Number of learners: not specified Healthcare professionals: acute stroke care (interdisciplinary team) Hospital: emergency department	Simulation training intervention—details not specified	Not specified	Door-to-needle time	11.5
Bohmann et al. 2022 [63] Germany	Number of patients: • Pre-simulation training: 175 patients • Post-simulation training: 169 patients Number of learners: 186 Healthcare professionals: Stroke team [40% residents, 7% specialist physicians, 18% senior physicians, 20% nurses, 4% students, 6% others (laboratory and radiology technicians)] Hospitals: Seven tertiary care neuro-centres in emergency departments of university hospitals	Theoretical introductions based on Crew Resource Management which was the stroke teams’ basis for the in-situ simulation. Simulation was structured as briefing → simulation → debriefing Simulation training of scripted simulation scenarios used high-fidelity manikins with a monitoring system to provide the cardiorespiratory alarms and standardise the simulation to focus on team communication. CT scans were used to present radiological findings	Two full days of stroke team training at each of the seven sites lasting approximately 3 h. No participant received more than one training	Door-to-needle time^a^ Self-perceived usefulness of simulation training	13.0
Rhew et al. 2022 [64] USA	Number of patients: not specified Number of learners: not specified Healthcare professionals: emergency department nurses Hospital: simulation lab	At the simulation skills fairs in the simulation lab, stroke simulation scenarios were performed by groups of nurses in the care of code stroke patients (details not specified). The simulation was facilitated by an experienced emergency department nurse	Emergency Department nurses were required to attend one of five simulation skills fairs offered over a 9-month period	Door-to-needle time	9.5
Svobodova et al. 2023 [65] Czech Republic	Number of patients: • Pre-simulation training: 14,046 patients • Post-simulation training: 3088 patients Number of learners: 94 Healthcare professionals: 62 physicians (mostly neurologists) and 32 nurses Hospital: equipped simulation centres	Two rounds of simulation scenarios (briefing → simulation → debriefing → conclusion) Scenarios were based on real life thrombolytic cases adapted for educational purposes, with hospital staff acting as simulated patients	Half-day simulation training spread over 10 courses	Door-to-needle time^a^ Self-perceived usefulness of simulation training Improvement in communication	12.5

*Abbreviations*:

*MERSQI* Medical Education Research Study Quality Instrument,

*PGY* Postgraduate Year in US residency training programmes

^a^Door-to-needle times were obtained from hospital stroke/thrombolysis registry

### Outcomes

The outcomes are divided into primary and secondary outcomes.

#### Primary outcome

##### Door-to-needle time

**Fig. 2:**
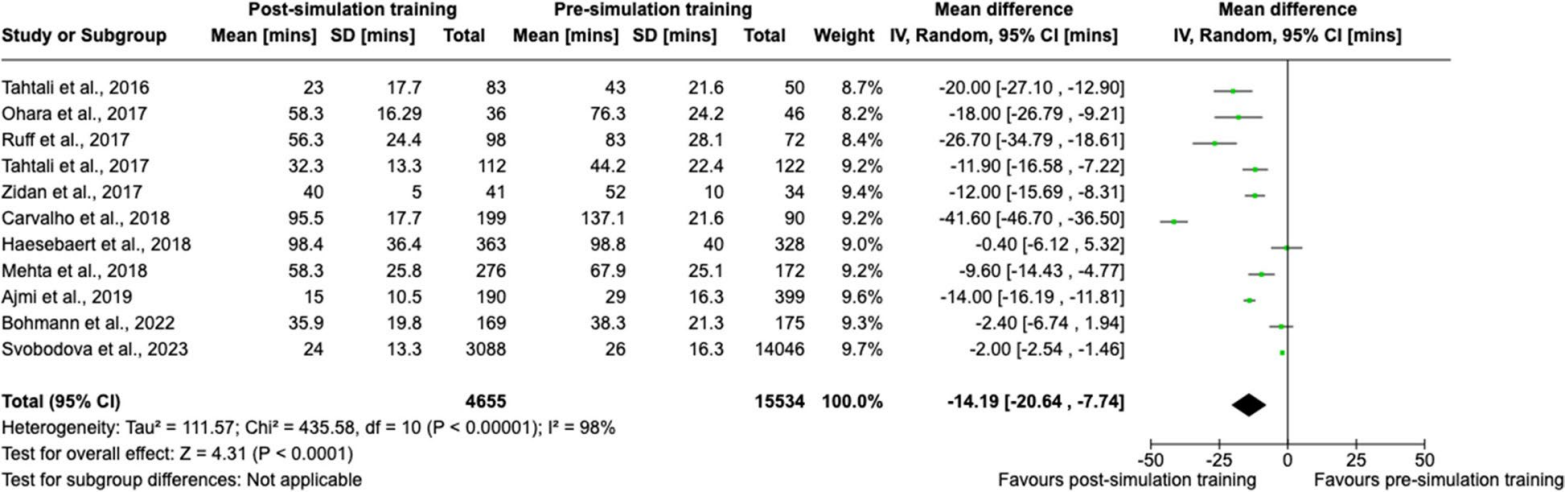
Random-effects meta-analysis assessing door-to-needle time (mins) pre- and post-simulation training

Eleven studies were eligible for meta-analysis on door-to-needle time (*n* = 20,189 patients) [47, 49, 51, 52, 55–58, 60, 63, 65]. Meta-analysis showed a statistically significant effect favouring post-simulation training in reducing door-to-needle time compared to pre-simulation training, with a pooled effect size of − 14.2 (95% CI − 20.6, − 7.7) (Fig. 2). The heterogeneity was high (*I*^*2*^ = 98%). The quality of studies ranged from 11.5 to 14.5 on the MERSQI scale.

The studies not included in the meta-analysis due to incomplete data [48, 50, 53, 54, 59, 61, 62, 64] individually showed reduced door-to-needle times post-simulation training. Three studies reported median reductions to 54 [59], 51 [54], and 32 min [62], respectively, and four studies reported mean reductions of 17 [48], 11 [64], 9.7 [50], and 9 min [53], respectively. In addition, one study reported a 100% improvement post-simulation [61]. The quality of studies ranged from 9.0 to 12.0 on the MERSQI scale.

### Sensitivity analysis

**Fig. 3:**
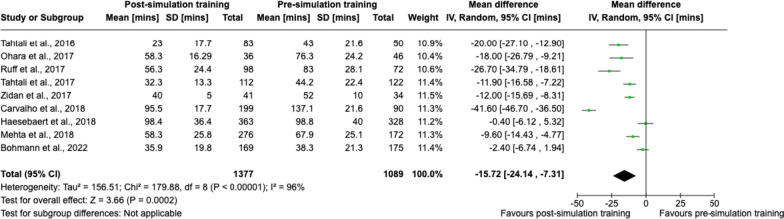
Sensitivity analysis using a random-effects meta-analysis for studies with low-to-moderate risk of bias

Sensitivity analysis was performed for the primary outcome of door-to-needle time in nine studies with an overall low-to-moderate risk of bias [47, 49, 51, 52, 55–58, 63]. Meta-analysis results remained consistent with a statistically significant pooled effect size favouring post-simulation training, mean difference − 15.7 (95% CI − 24.1 to − 7.3) min (Fig. 3).

**Fig. 4:**
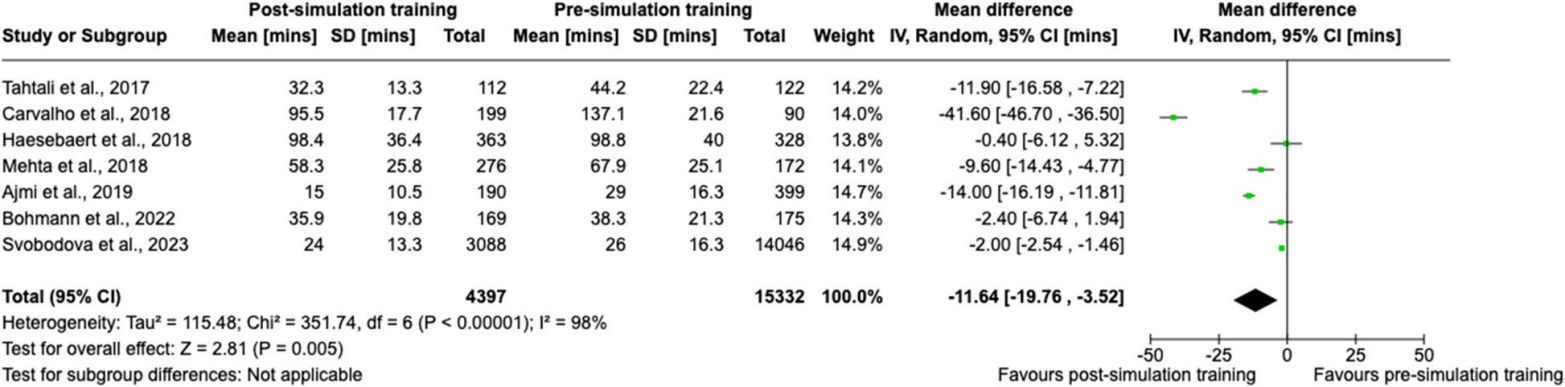
Sensitivity analysis using the random-effects meta-analysis for studies with moderate-to-high methodological quality

A sensitivity analysis was also performed in seven studies [52, 56–58, 60, 63, 65] of moderate-to-high methodological quality on the MERSQI scale (≥ 12.5 out of 18), also with a statistically significant pooled effect in favour of post-simulation training, mean difference − 11.6 (95% CI − 19.8 to − 3.5) min (Fig. 4).

#### Secondary outcomes

##### Improvement in acute stroke knowledge

**Fig. 5:**
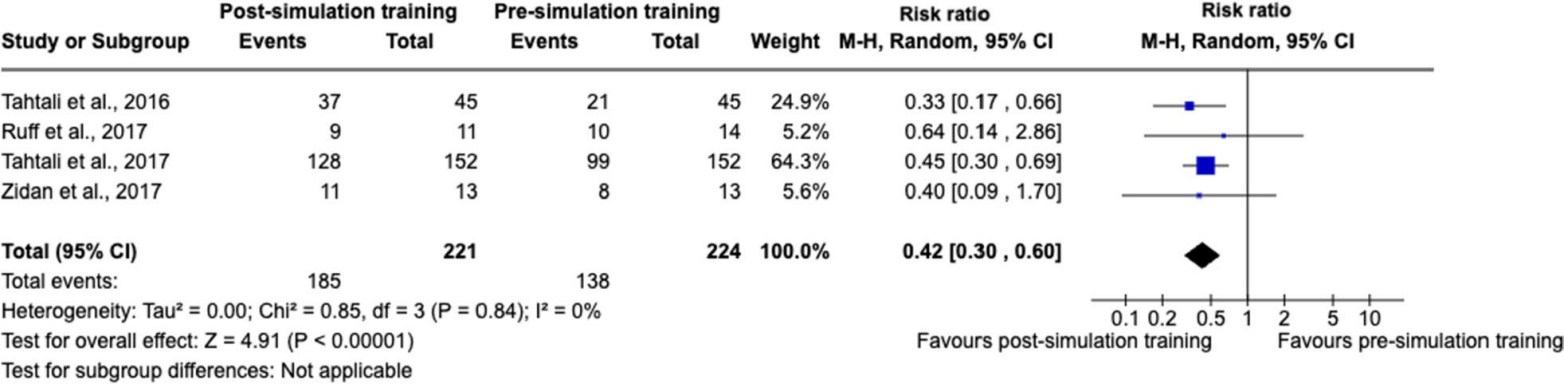
Random-effects meta-analysis of improvement in acute stroke knowledge pre- and post-simulation training

Four studies [47, 51, 52, 55] assessed improvement in knowledge through surveys, reporting improvements ranging from 46.6% pre-simulation training to 84.6% post-simulation training. Meta-analysis showed a statistically significant effect in favour of post-simulation training in improving healthcare professionals’ acute stroke knowledge, with a pooled RR of 0.42 (95% CI 0.30 to 0.60) (Fig. 5).

##### Feeling ‘safe’ in thrombolysis-related decision-making

**Fig. 6:**
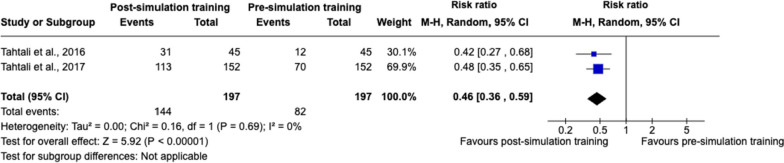
Random-effects meta-analysis of healthcare professionals feeling ‘safe’ in thrombolysis-related decision-making

Two studies [47, 52] assessed healthcare professionals' feelings of ‘safety’ in thrombolysis-related decision-making, reporting improvements ranging from 26.7 to 74.3%. Meta-analysis showed a statistically significant effect favouring post-simulation training with respect to improved feelings of safety in thrombolysis-related decision-making, with a pooled RR of 0.46 (95% CI 0.36 to 0.59) (Fig. 6).

##### Self-perceived usefulness of simulation training

Four studies assessed the self-perceived usefulness of simulation training using Likert scales, showing relatively high scores of 95.5% [63], 90.0% [60], 88.4% [52], and 85.0% [65], respectively (Table 3A). The average improvement score was 89.7%. No pre-simulation data was available; therefore, meta-analysis was not possible.

**Table 3:** Secondary outcome measures post-simulation training (A) perceived usefulness of training (B) improvement in communication

(A)	Author, year	Perceived usefulness of training (post-simulation training assessment) % that rated useful
	Tahtali et al. 2017 [52]	88.4%
	Ajmi et al. 2019 [60]	90.0%
	Bohmann et al. 2022 [63]	95.5%
	Svobodova et al. 2023 [65]	85.0%
(B)	Author, year	Improvement in communication (post-simulation training assessment)
	Tahtali et al. 2016 [47]	Improved to 90.0%
	Svobodova et al. 2023 [65]	Improved to 77.0%

Two studies [47, 63] expressed interest in regular simulation sessions; one reported that 93.6% of participants would welcome annual training [63], another desired annual (46.0%) and semi-annual (49.0%) repetition of training [47].

##### Improvement in communication

Two studies [47, 65] assessed improvements in communication post-simulation, reporting 90.0% [47] and 77.0% [65] improvement (Table 3B). No pre-simulation data was available; therefore, meta-analysis was not possible.

### Publication bias

**Fig. 7:**
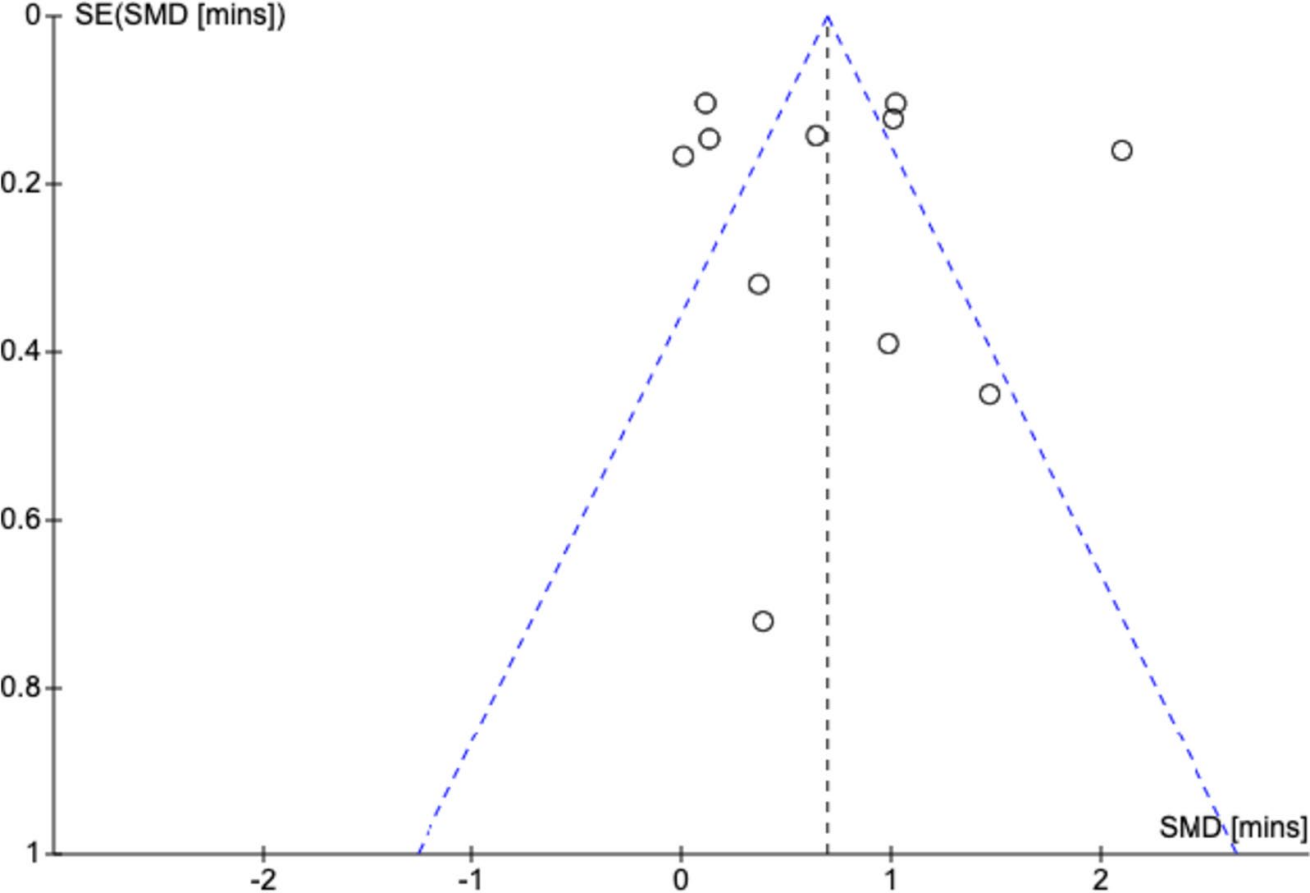
Publication bias of eleven studies included in the meta-analysis of door-to-needle time

A Funnel plot of the eleven studies included in the meta-analysis with respect to door-to-needle time was generated. Visual inspection reveals asymmetry (Fig. 7).

### Risk of Bias (ROBINS-I) in included studies

**Fig. 8:**
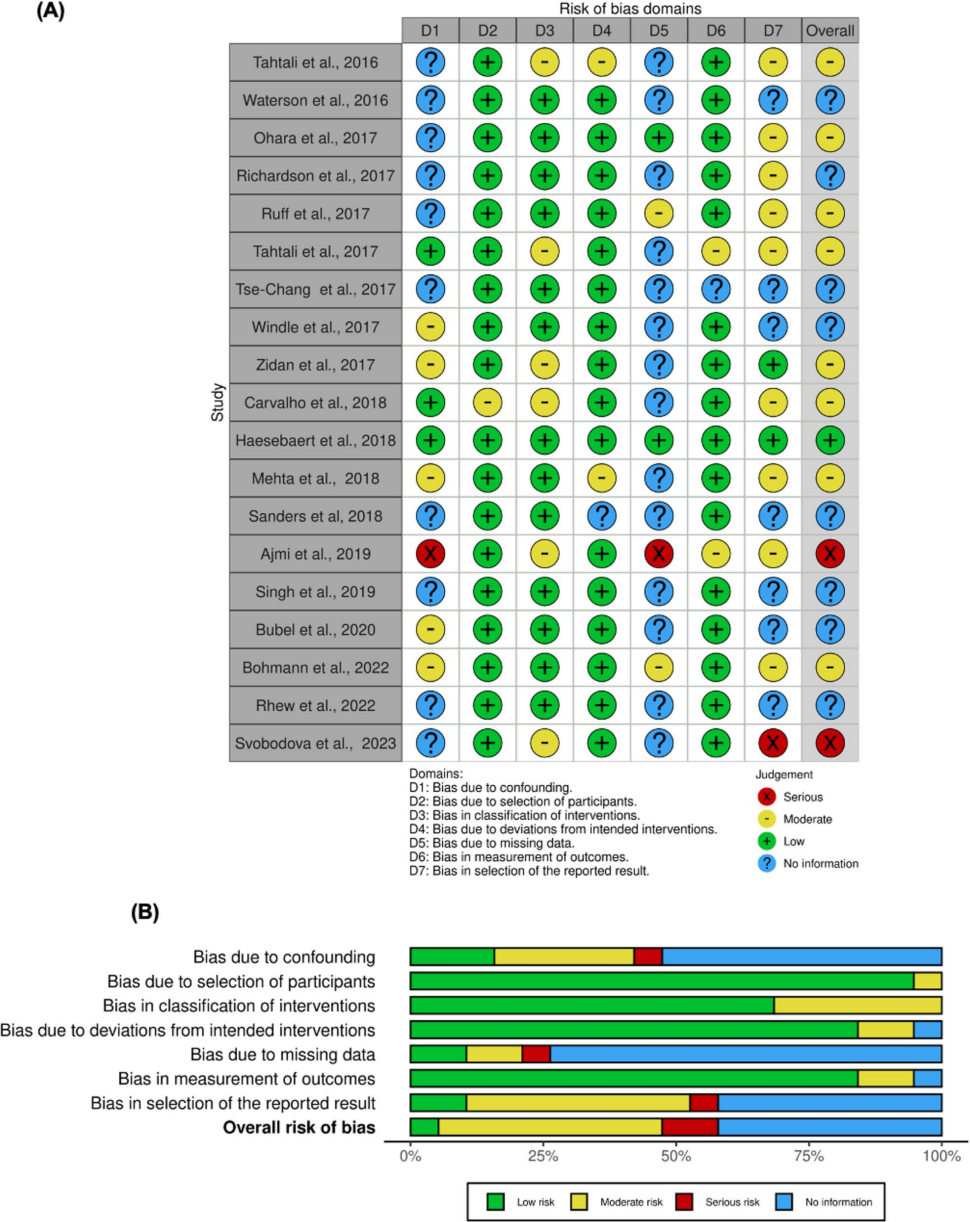
Risk of bias (ROBINS-I) of studies included in the meta-analysis

The risk of bias was assessed using a ROBINS-I risk-of-bias graph (Fig. 8A, B). Most studies had good participant selection of healthcare professionals and outcome measurements, with low deviations from intended interventions. Bias from confounding, missing data, and selective reporting of results were difficult to assess due to insufficient information. One study had an overall low risk of bias [57], eight had a moderate risk of bias [47, 49, 51, 52, 55, 56, 58, 63], two had a serious risk of bias [60, 65], and eight had insufficient information [48, 50, 53, 54, 59, 61, 62, 64].

### Heterogeneity

No studies were excluded based on methodological heterogeneity. There was a high estimate of statistical heterogeneity (*I*^*2*^ = 98%) in those studies included in our primary analysis with respect to door-to-needle time for intravenous thrombolysis in ischaemic stroke patients (Fig. 2).

## Discussion

In this systematic review and meta-analysis, we found that simulation had a beneficial effect, reducing door-to-needle time for the emergency delivery of intravenous thrombolysis in patients with ischaemic stroke by about 15 min. For each minute in a middle cerebral artery stroke without treatment, around 1.9 million neurons are lost [68]. Each 15-min reduction in delay to treatment may achieve a 4% increase in good clinical outcomes [69]. As such, numerous guidelines are advocating for a downward revision in door-to-needle target times, aiming for under 30 min [70]. Given the well-established relationship between early recombinant tissue plasminogen activator (rtPA) administration and improved patient outcomes [70], an improvement of this magnitude has important clinical implications.

Despite notable methodological and statistical heterogeneity among the included studies, sensitivity analysis corroborated the collective impact favouring post-simulation training. Methodological variances arose from several sources. The studies were conducted across ten different countries, each with particular healthcare system nuances. Though international stroke care standards are recognised, these may be harder to fulfil in some settings than others, and the areas for gain with regard to improving door-to-needle times may vary greatly. For example, delays may be apparent to a greater or lesser extent at different points in the patient journey, dependent on resources such as a dedicated stroke physician or access to computed tomography (CT) scanning. Such variations would be challenging to control for, even in multicentre randomised controlled trials. Moreover, study participants varied significantly in terms of numbers and healthcare professions, including physicians of various levels of experience, from stroke care specialists to radiologists, paramedics, and nurses. There was also diversity in the approaches to conducting simulations, both in terms of the environments the simulations were conducted in, ranging from in situ simulations to classroom-based scenarios, and how patients were represented within the simulations, ranging from patient actors to manikins.

While implementing simulation training, five studies concurrently revised their stroke protocol, raising the possibility of validation bias, and three used multifaceted interventions, increasing confounding and difficulty attributing complete improvements in door-to-needle time to simulation training alone. However, simulation training cannot be considered a stand-alone activity in itself but rather one part of a multi-faceted approach to quality improvement, systems redesign and testing, and team/organisational culture [71, 72]. Translational simulation for transformative rather than pedagogical purposes is being recognised as a growing field as a means to promote change within clinical systems [73–75]. Considering that simulation training is an entanglement of activities in process and quality improvement, such as testing new protocols, a different sensitivity analysis, and separating studies with concurrent interventions, was not conducted.

Despite the explained methodological diversity across the studies, there was adequate consistency in reported outcomes related to door-to-needle time and learner experiences to synthesise the results. Our findings align with existing literature on the application of simulation training to enhance aspects of care in other emergency patient scenarios, such as cardiac arrest [76], cardiac catheterisation [77], extracorporeal membrane oxygenation [78], and maternal cardiac arrest [79].

With respect to our learner-centred secondary outcomes, we found that stroke subject knowledge, clinical perception of safety in thrombolysis decision-making, self-perceived usefulness, and communication were all found to increase after simulation training, mirroring the existing literature from other clinical environments [80–82]. Drawing a direct causative relationship is not possible for these qualitative outcomes, but it seems likely that the suitability of simulation training for understanding and improving macro-ergonomics and human factors is important [83].

The successful and timely administration of intravenous thrombolysis involves highly complex systems and multidisciplinary teams. Expert consensus acknowledges simulation training as particularly beneficial for testing, practising, executing, and evaluating peri-operative microsystems [84]. These microsystems are also high-risk, complex hospital systems involving large multidisciplinary teams caring for patients having surgical procedures, and our findings support the possibility that this also applies to acute stroke management. Additionally, simulation training is well-recognised for its benefits in multidisciplinary team education [85, 86], which may contribute to our findings of an overall reduction in door-to-needle time. Simulation training in neurocritical care has been slower to gain acceptance compared to other medical disciplines [87]. This may be partly due to the challenge of replicating time-sensitive neurological emergencies like stroke and status epilepticus using simulation manikins or actors [86, 87].

Kirkpatrick’s model for evaluation of training is a four-step model which categorises learning outcomes into four levels: (1) reaction, (2) learning, (3) behaviour, and (4) results [88]. In clinical environments, Level 4 outcomes can be represented by patient-centred outcomes. This review implies that both Level 2 (learner-reported outcomes) and Level 4 outcomes (door-to-needle time) align with Kirkpatrick’s scale. A recent systematic review that covered a wide range of medical education simulations only identified 13 studies for inclusion and reported a paucity of studies employing Kirkpatrick’s Level 4 outcomes to evaluate simulation training [89].

While the overall quality of the studies included in this review is, at best, moderate, it is worth noting that this study is not the first to question the quality of studies in simulation training. Even when reviewing randomised controlled trials of simulations [90], authors found a high risk of bias (86%), a lack of reported findings (4%), an absence of registered protocols, as well as various issues with blinding and concealment. While simulation training appears to be a valuable technique in various aspects of medical education, there is sufficient equipoise for further high-quality, standardised studies evaluating Kirkpatrick’s Level 4 outcomes.

## Strengths

This meta-analysis is the first to assess the effects of simulation training on door-to-needle time regarding emergency thrombolysis delivery to patients with ischaemic stroke and has important strengths. First, it was conducted according to PRISMA guidelines [32] and encompassed a comprehensive search strategy with no language restrictions. Second, the authors of all included studies with missing data were contacted regarding the existence of any other data to ensure methodological robustness. Third, methodological data extraction utilised a custom-created extraction sheet. Fourth, the ROBINS-I tool was applied to accurately assess the risk of bias, facilitating the estimation of the true effects of simulation training [44]. Lastly, two sensitivity analyses were performed to ensure the quality and robustness of the results [91].

## Limitations

The limitations of this meta-analysis primarily pertain to the weaknesses of the source articles. First, the limited number of studies and variance in methods (only one was a randomised control study), sample sizes, study periods, and number of strokes may influence the validity of the findings. We found a high degree of heterogeneity among the included studies, and though we used a random-effects model to account for this, we recognise this as a weakness, affecting the strength of our conclusions. Second, the concurrent introduction of simulation training and revision of stroke protocols in some studies raises the possibility of validation bias. While this is important for meta-analysis, simulation training is not a stand-alone activity and, therefore, must be interpreted within the context of quality improvement. Third, the critical appraisal using the ROBINS-I tool was performed by one independent reviewer, which may have increased the risk of bias. Fourth, the asymmetry of the funnel plot indicates heterogeneity and publication bias among the included studies. Fifth, translational simulation was not included as a search term, and therefore, the search strategy may have failed to capture evidence on this aspect. Sixth, the reported medians and interquartile ranges (IQR) were converted to means and standard deviations (SD), respectively, which may have introduced bias and imprecise estimates. Seventh, a sensitivity analysis separating studies with concurrent interventions was not performed due to simulation training being an entanglement of activities in process and quality improvements, which may have increased the risk of bias when assessing the pure effect of simulation. Lastly, we acknowledge that this review was not registered with PROSPERO.

## Future directions

There is a need for robust and standardised multi-institutional studies with randomised controlled trial designs using Kirkpatrick’s Level 4 outcomes and larger sample sizes, inclusive of all healthcare professionals involved in the delivery of emergency thrombolysis in ischaemic stroke. Future studies should standardise the reporting of simulation-based interventions using standardised reporting tools [41], clearly demarcating its different types using rigorous and reproducible outcome measures, namely the Kirkpatrick Model [92]. Future work should consider that simulation for healthcare improvement is one part of any contemporary quality improvement strategy, and as such, the results of simulation-based studies need to be interpreted and considered alongside simultaneous contextual changes such as protocol refinements or process changes within complex systems.

## Conclusion

This meta-analysis showed a significant beneficial effect of simulation training in reducing door-to-needle time delivery of emergency thrombolysis in ischaemic stroke. Additionally, simulation training was associated with improved knowledge, communication, and a feeling of ‘safety’ in thrombolysis-related decision-making. The results should be interpreted with caution due to the heterogeneity of the included studies. Further high-quality research is warranted to strengthen the evidence base and establish confidence in the effect measures.

### Supplementary Information

**Additional file 1: Appendix 1.** PRISMA 2020 Checklist. **Appendix 2.** Search strategies. **Appendix 3.** Sample data extraction sheet. **Appendix 4.** Details on the assessment of risk of bias ROBINS-I results. **Appendix 5.** Table of excluded studies.

## Data Availability

Data is available upon request.

## Abbreviations

CT: Computed tomography
CI: Confidence interval
ED: Emergency department
FAST: Facial drooping, Arm weakness, Speech difficulties and Time
IQR: Interquartile range
MERSQI: Medical Education Research Study Quality Instrument
PICO: Participants, Intervention, Comparisons and Outcomes
PRISMA: Preferred Reporting Items for Systematic Review and Meta-Analysis
rtPA: Recombinant tissue plasminogen activator
RevMan: Review Manager
ROBINS-I: Risk of Bias in Non-Randomised Studies of Interventions
RR: Risk ratio
SD: Standard deviation
UK: United Kingdom
USA: United States of America
