# Supplementary material for: Evaluating the effects of simulation training on stroke thrombolysis: a systematic review and meta-analysis
Source: Adv Simul (Lond). 2024 Feb 29;9:11. doi: 10.1186/s41077-024-00283-6 (PMC10905914; doi:10.1186/s41077-024-00283-6)
Supplement: Supplementary file 1 — Additional file 1: Appendix 1. PRISMA 2020 Checklist. Appendix 2. Search strategies. Appendix 3. Sample data extraction sheet. Appendix 4. Details on the assessment of risk of bias ROBINS-I results. Appendix 5. Table of excluded studies. [file 41077_2024_283_MOESM1_ESM.docx]

**APPENDICES**

**APPENDIX 1 – PRISMA 2020 Checklist**

| **Section and Topic** | **Item #** | **Checklist item** | **Location where item is reported** |
| --- | --- | --- | --- |
| **TITLE** | | |  |
| Title | 1 | Identify the report as a systematic review. | Title page |
| **ABSTRACT** | | |  |
| Abstract | 2 | See the PRISMA 2020 for Abstracts checklist. | Abstract |
| **INTRODUCTION** | | |  |
| Rationale | 3 | Describe the rationale for the review in the context of existing knowledge. | Background |
| Objectives | 4 | Provide an explicit statement of the objective(s) or question(s) the review addresses. | Aim and objectives |
| **METHODS** | | |  |
| Eligibility criteria | 5 | Specify the inclusion and exclusion criteria for the review and how studies were grouped for the syntheses. | Methods Table 1 |
| Information sources | 6 | Specify all databases, registers, websites, organisations, reference lists and other sources searched or consulted to identify studies. Specify the date when each source was last searched or consulted. | Methods Study Identification |
| Search strategy | 7 | Present the full search strategies for all databases, registers and websites, including any filters and limits used. | Appendix 1 |
| Selection process | 8 | Specify the methods used to decide whether a study met the inclusion criteria of the review, including how many reviewers screened each record and each report retrieved, whether they worked independently, and if applicable, details of automation tools used in the process. | Methods Study Identification and Selection |
| Data collection process | 9 | Specify the methods used to collect data from reports, including how many reviewers collected data from each report, whether they worked independently, any processes for obtaining or confirming data from study investigators, and if applicable, details of automation tools used in the process. | Methods Data Extraction |
| Data items | 10a | List and define all outcomes for which data were sought. Specify whether all results that were compatible with each outcome domain in each study were sought (e.g. for all measures, time points, analyses), and if not, the methods used to decide which results to collect. | Methods Data Synthesis |
|  | 10b | List and define all other variables for which data were sought (e.g. participant and intervention characteristics, funding sources). Describe any assumptions made about any missing or unclear information. | Methods Study Eligibility and Table 1 |
| Study risk of bias assessment | 11 | Specify the methods used to assess risk of bias in the included studies, including details of the tool(s) used, how many reviewers assessed each study and whether they worked independently, and if applicable, details of automation tools used in the process. | Methods Assessment of Risk of Bias and Study Quality |
| Effect measures | 12 | Specify for each outcome the effect measure(s) (e.g. risk ratio, mean difference) used in the synthesis or presentation of results. | Methods Data Synthesis |
| Synthesis methods | 13a | Describe the processes used to decide which studies were eligible for each synthesis (e.g. tabulating the study intervention characteristics and comparing against the planned groups for each synthesis (item #5)). | Methods Data Exraction |
|  | 13b | Describe any methods required to prepare the data for presentation or synthesis, such as handling of missing summary statistics, or data conversions. | Methods Data Extraction |
|  | 13c | Describe any methods used to tabulate or visually display results of individual studies and syntheses. | Methods Data Extraction |
|  | 13d | Describe any methods used to synthesize results and provide a rationale for the choice(s). If meta-analysis was performed, describe the model(s), method(s) to identify the presence and extent of statistical heterogeneity, and software package(s) used. | Methods Data Synthesis |
|  | 13e | Describe any methods used to explore possible causes of heterogeneity among study results (e.g. subgroup analysis, meta-regression). | Not performed |
|  | 13f | Describe any sensitivity analyses conducted to assess robustness of the synthesized results. | Methods Data Synthesis (Sensitivity Analysis) |
| Reporting bias assessment | 14 | Describe any methods used to assess risk of bias due to missing results in a synthesis (arising from reporting biases). | Methods Assessment of Risk of Bias and Study Quality |
| Certainty assessment | 15 | Describe any methods used to assess certainty (or confidence) in the body of evidence for an outcome. | Methods Assessment of Risk of Bias and Study Quality |
| **RESULTS** | | |  |
| Study selection | 16a | Describe the results of the search and selection process, from the number of records identified in the search to the number of studies included in the review, ideally using a flow diagram. | Results Figure 1 PRISMA flow diagram |
|  | 16b | Cite studies that might appear to meet the inclusion criteria, but which were excluded, and explain why they were excluded. | Appendix 3 |
| Study characteristics | 17 | Cite each included study and present its characteristics. | Results Table 2 |
| Risk of bias in studies | 18 | Present assessments of risk of bias for each included study. | Results, Risk of bias (ROBINS-I) in Included Studies |
| Results of individual studies | 19 | For all outcomes, present, for each study: (a) summary statistics for each group (where appropriate) and (b) an effect estimate and its precision (e.g. confidence/credible interval), ideally using structured tables or plots. | Results |
| Results of syntheses | 20a | For each synthesis, briefly summarise the characteristics and risk of bias among contributing studies. | Results Risk of bias (ROBINS-I) in Included Studies |
|  | 20b | Present results of all statistical syntheses conducted. If meta-analysis was done, present for each the summary estimate and its precision (e.g. confidence/credible interval) and measures of statistical heterogeneity. If comparing groups, describe the direction of the effect. | Results, Primary outcome and Secondary outcomes |
|  | 20c | Present results of all investigations of possible causes of heterogeneity among study results. | Results |
|  | 20d | Present results of all sensitivity analyses conducted to assess the robustness of the synthesized results. | Results Sensitivity Analysis |
| Reporting biases | 21 | Present assessments of risk of bias due to missing results (arising from reporting biases) for each synthesis assessed. | Results Study Characteristics and Risk of Bias |
| Certainty of evidence | 22 | Present assessments of certainty (or confidence) in the body of evidence for each outcome assessed. | Results Table 2. Study Characteristics MERSQI |
| **DISCUSSION** | | |  |
| Discussion | 23a | Provide a general interpretation of the results in the context of other evidence. | Discussion |
|  | 23b | Discuss any limitations of the evidence included in the review. | Discussion Limitations Section |
|  | 23c | Discuss any limitations of the review processes used. | Discussion Limitations Section |
|  | 23d | Discuss implications of the results for practice, policy, and future research. | Discussion Future Directions |
| **OTHER INFORMATION** | | |  |
| Registration and protocol | 24a | Provide registration information for the review, including register name and registration number, or state that the review was not registered. | Abstract |
|  | 24b | Indicate where the review protocol can be accessed, or state that a protocol was not prepared. | Protocol was not prepared |
|  | 24c | Describe and explain any amendments to information provided at registration or in the protocol. | N/A |
| Support | 25 | Describe sources of financial or non-financial support for the review, and the role of the funders or sponsors in the review. | Funding Section |
| Competing interests | 26 | Declare any competing interests of review authors. | Competing Interests |
| Availability of data, code and other materials | 27 | Report which of the following are publicly available and where they can be found: template data collection forms; data extracted from included studies; data used for all analyses; analytic code; any other materials used in the review. | Data Availability Section |

**APPENDIX 2 – Search Strategies**

**Database: Embase** <1990 to 2023 Week 19

| **#** | **Query** | **Results from 17 May 2023** |
| --- | --- | --- |
| 1 | simulation training.mp. or exp Simulation Training/ or exp Patient Simulation/ | 13,389 |
| 2 | exp Computer Simulation/ or exp Manikins/ or exp High Fidelity Simulation Training/ or exp Computer-Assisted Instruction/ | 268,069 |
| 3 | (simulat* adj5 (educat* or cours* or workshop* or boot-camp* or bootcamp* or learn* or experienc* or curricul* or framework* or teach* or guid* or coach* or supervis* or seminar* or lectur* or round* or tutor* or mentor* or program* or training* or event*)).mp. | 58,887 |
| 4 | (high adj3 fidelity adj5 (educat* or cours* or workshop* or boot-camp* or bootcamp* or learn* or experienc* or curricul* or framework* or teach* or guid* or coach* or supervis* or seminar* or lectur* or round* or tutor* or mentor* or program* or training* or event*)).mp. | 2,663 |
| 5 | (hi-fi* adj5 (educat* or cours* or workshop* or boot-camp* or bootcamp* or learn* or experienc* or curricul* or framework* or teach* or guid* or coach* or supervis* or seminar* or lectur* or round* or tutor* or mentor* or program* or training* or event*)).mp. | 29 |
| 6 | (in adj3 situ adj5 (educat* or cours* or workshop* or boot-camp* or bootcamp* or learn* or experienc* or curricul* or framework* or teach* or guid* or coach* or supervis* or seminar* or lectur* or round* or tutor* or mentor* or program* or training* or event*)).mp. | 4,183 |
| 7 | (insitu adj5 (educat* or cours* or workshop* or boot-camp* or bootcamp* or learn* or experienc* or curricul* or framework* or teach* or guid* or coach* or supervis* or seminar* or lectur* or round* or tutor* or mentor* or program* or training* or event*)).mp. | 24 |
| 8 | (full adj3 scale* adj5 (educat* or cours* or workshop* or boot-camp* or bootcamp* or learn* or experienc* or curricul* or framework* or teach* or guid* or coach* or supervis* or seminar* or lectur* or round* or tutor* or mentor* or program* or training* or event*)).mp. | 569 |
| 9 | (manikin* or mannequin*).tw. | 8,537 |
| 10 | ((virtural or technology) adj3 simulation*).tw. | 1,281 |
| 11 | 1 or 2 or 3 or 4 or 5 or 6 or 7 or 8 or 9 or 10 | 320,409 |
| 12 | exp Thrombolytic therapy/ | 29,435 |
| 13 | Thrombolysis.tw. | 47,976 |
| 14 | exp Fibrinolysis/ | 79,166 |
| 15 | exp Tissue Plasminogen Activator/ or exp Plasminogen Activators/ | 88,781 |
| 16 | Fibrinolytic agents/ or Plasmin/ or Plasminogen/ | 42,650 |
| 17 | (thromboly$ or fibrinoly$ or clot lysis).tw. | 103,567 |
| 18 | (plasminogen or plasmin or tPA or t-PA or rtPA or rt-PA).tw. | 91,340 |
| 19 | (anistreplase or tenecteplase or streptodornase or streptokinase or urokinase or pro?urokinase or rpro?uk or lumbrokinase or duteplase or lanoteplase or pamiteplase or reteplase or saruplase or staphylokinase or streptase or alteplase).tw. | 32,152 |
| 20 | Thrombosis/dt [Drug Therapy] | 18,218 |
| 21 | Thrombolytic therapy methods.tw. | 54 |
| 22 | (Door to needle or dtn door-to-thrombolysis or in-hospital delay* or onset-to-treatment or interval time* or triage-to-treatment or treatment time or tPA time).tw. | 32,936 |
| 23 | exp Time-to-Treatment/ | 25,700 |
| 24 | 12 or 13 or 14 or 15 or 16 or 17 or 18 or 19 or 20 or 21 or 22 or 23 | 293,241 |
| 25 | stroke.mp. or exp Stroke/ | 628,571 |
| 26 | Stroke*.tw. | 494,939 |
| 27 | exp Ischemic Stroke/ | 25,154 |
| 28 | Acute stroke.tw. | 33,661 |
| 29 | exp Cerebrovascular Disorders/ | 795,542 |
| 30 | exp Ischemic Attack, Transient/ | 45,558 |
| 31 | (Cerebrovascular accident or cerebrovascular event or cerebrovascular disease or CVA or CVE).tw. | 45,258 |
| 32 | 25 or 26 or 27 or 28 or 29 or 30 or 31 | 1,002,798 |
| 33 | patient care team.mp. or exp Patient Care Team/ | 999,371 |
| 34 | (Healthcare team or healthcare work* or health work* or healthcare provid* or healthcare personnel* or health care profession*).tw. | 155,274 |
| 35 | exp Critical Care/ | 829,913 |
| 36 | Intensive care.tw. | 274,001 |
| 37 | exp Intensive Care Units/ | 282,460 |
| 38 | stroke team.tw. | 1,298 |
| 39 | (multidisciplinary team or MDT).mp. | 60,553 |
| 40 | exp Physicians/ or Physicians.mp. | 1,192,698 |
| 41 | exp Nurses/ | 212,166 |
| 42 | Emergenc*.mp. or emergency department.tw. | 843,999 |
| 43 | (doctor adj1 (clinician* or radiolog* or practitioner or consultant or physician*)).tw. | 173 |
| 44 | (nurse adj1 practitioner).tw. | 8,702 |
| 45 | exp Radiologists/ | 68,361 |
| 46 | exp Paramedics/ or exp Allied Health Personnel/ | 579,425 |
| 47 | 33 or 34 or 35 or 36 or 37 or 38 or 39 or 40 or 41 or 42 or 43 or 44 or 45 or 46 | 3,853,196 |
| 48 | 11 and 24 and 32 and 47 | 208 |

**Database:** APA **PsycInfo** <1990 to May Week 2 2023>

| **#** | **Query** | **Results from 17 May 2023** |
| --- | --- | --- |
| 1 | simulation training.mp. or exp Simulation Training/ or exp Patient Simulation/ | 582 |
| 2 | exp Computer Simulation/ or exp Manikins/ or exp High Fidelity Simulation Training/ or exp Computer-Assisted Instruction/ | 43,440 |
| 3 | (simulat* adj5 (educat* or cours* or workshop* or boot-camp* or bootcamp* or learn* or experienc* or curricul* or framework* or teach* or guid* or coach* or supervis* or seminar* or lectur* or round* or tutor* or mentor* or program* or training* or event*)).mp. | 11,088 |
| 4 | (high adj3 fidelity adj5 (educat* or cours* or workshop* or boot-camp* or bootcamp* or learn* or experienc* or curricul* or framework* or teach* or guid* or coach* or supervis* or seminar* or lectur* or round* or tutor* or mentor* or program* or training* or event*)).mp. | 550 |
| 5 | (hi-fi* adj5 (educat* or cours* or workshop* or boot-camp* or bootcamp* or learn* or experienc* or curricul* or framework* or teach* or guid* or coach* or supervis* or seminar* or lectur* or round* or tutor* or mentor* or program* or training* or event*)).mp. | 2 |
| 6 | (in adj3 situ adj5 (educat* or cours* or workshop* or boot-camp* or bootcamp* or learn* or experienc* or curricul* or framework* or teach* or guid* or coach* or supervis* or seminar* or lectur* or round* or tutor* or mentor* or program* or training* or event*)).mp. | 312 |
| 7 | (insitu adj5 (educat* or cours* or workshop* or boot-camp* or bootcamp* or learn* or experienc* or curricul* or framework* or teach* or guid* or coach* or supervis* or seminar* or lectur* or round* or tutor* or mentor* or program* or training* or event*)).mp. | 0 |
| 8 | (full adj3 scale* adj5 (educat* or cours* or workshop* or boot-camp* or bootcamp* or learn* or experienc* or curricul* or framework* or teach* or guid* or coach* or supervis* or seminar* or lectur* or round* or tutor* or mentor* or program* or training* or event*)).mp. | 170 |
| 9 | (manikin* or mannequin*).tw. | 643 |
| 10 | ((virtural or technology) adj3 simulation*).tw. | 225 |
| 11 | 1 or 2 or 3 or 4 or 5 or 6 or 7 or 8 or 9 or 10 | 53,810 |
| 12 | exp Thrombolytic therapy/ | 0 |
| 13 | Thrombolysis.tw. | 1,200 |
| 14 | exp Fibrinolysis/ | 0 |
| 15 | exp Tissue Plasminogen Activator/ or exp Plasminogen Activators/ | 0 |
| 16 | Fibrinolytic agents/ or Plasmin/ or Plasminogen/ | 0 |
| 17 | (thromboly$ or fibrinoly$ or clot lysis).tw. | 1,603 |
| 18 | (plasminogen or plasmin or tPA or t-PA or rtPA or rt-PA).tw. | 1,586 |
| 19 | (anistreplase or tenecteplase or streptodornase or streptokinase or urokinase or pro?urokinase or rpro?uk or lumbrokinase or duteplase or lanoteplase or pamiteplase or reteplase or saruplase or staphylokinase or streptase or alteplase).tw. | 327 |
| 20 | [Thrombosis/dt [Drug Therapy]] | 0 |
| 21 | Thrombolytic therapy methods.tw. | 1 |
| 22 | (Door to needle or dtn door-to-thrombolysis or in-hospital delay* or onset-to-treatment or interval time* or triage-to-treatment or treatment time or tPA time).tw. | 1,497 |
| 23 | exp Time-to-Treatment/ | 0 |
| 24 | 12 or 13 or 14 or 15 or 16 or 17 or 18 or 19 or 20 or 21 or 22 or 23 | 4,031 |
| 25 | stroke.mp. or exp Stroke/ | 40,731 |
| 26 | Stroke*.tw. | 39,271 |
| 27 | exp Ischemic Stroke/ | 0 |
| 28 | Acute stroke.tw. | 2,299 |
| 29 | exp Cerebrovascular Disorders/ | 31,536 |
| 30 | exp Ischemic Attack, Transient/ | 0 |
| 31 | (Cerebrovascular accident or cerebrovascular event or cerebrovascular disease or CVA or CVE).tw. | 3,727 |
| 32 | 25 or 26 or 27 or 28 or 29 or 30 or 31 | 48,994 |
| 33 | patient care team.mp. or exp Patient Care Team/ | 8,394 |
| 34 | (Healthcare team or healthcare work* or health work* or healthcare provid* or healthcare personnel* or health care profession*).tw. | 30,967 |
| 35 | exp Critical Care/ | 0 |
| 36 | Intensive care.tw. | 10,042 |
| 37 | exp Intensive Care Units/ | 0 |
| 38 | stroke team.tw. | 53 |
| 39 | (multidisciplinary team or MDT).mp. | 3,316 |
| 40 | exp Physicians/ or Physicians.mp. | 74,944 |
| 41 | exp Nurses/ | 34,986 |
| 42 | Emergenc*.mp. or emergency department.tw. | 71,308 |
| 43 | (doctor adj1 (clinician* or radiolog* or practitioner or consultant or physician*)).tw. | 32 |
| 44 | (nurse adj1 practitioner).tw. | 1,507 |
| 45 | exp Radiologists/ | 0 |
| 46 | exp Paramedics/ or exp Allied Health Personnel/ | 6,417 |
| 47 | 33 or 34 or 35 or 36 or 37 or 38 or 39 or 40 or 41 or 42 or 43 or 44 or 45 or 46 | 219,788 |
| 48 | 11 and 24 and 32 and 47 | 3 |

**Database:** EBSCO **(ERIC)** Wednesday, May 17, 2023, 2:55:29 PM

| **#** | **Query** | **Results** |
| --- | --- | --- |
| **S1** | (MH "Simulations+") OR (MH "Computer Simulation+") OR (MH "Computerized Clinical Simulation Testing") OR (MH "Patient Simulation") OR "simulation training or simulation education or simulation learning" | 0 |
| **S2** | manikin simulation or manikins or manneqins or human anatomical models or simulation | 31 |
| **S3** | high fidelity simulation training | 11 |
| **S4** | virtual technology simulation | 29 |
| **S5** | high fidelity workshop or bootcamp or course | 10 |
| **S6** | Insitu simulation or workshop or bootcamp or course | 10 |
| **S7** | S1 OR S2 OR S3 OR S4 OR S5 OR S6 | 109 |
| **S8** | thrombolytic treatment or thombolysis or tissue plasminogen activator or tpa or alteplase | 1 |
| **S9** | thrombolytic therapy methods | 1 |
| **S10** | (MH "Thrombolytic Therapy") OR "thrombolysis or thrombolytic therapy or fibrinolysis or fibrinolytic therapy" | 1 |
| **S11** | plasminogen activators or tpa or t-pa or rtpa or rt-pa | 2 |
| **S12** | alteplase or tenectaplase | 0 |
| **S13** | door to needle time or door to needle time stroke or dtn or door to thrombolysis | 1 |
| **S14** | onset to treatment or time to treatment | 8 |
| **S15** | S8 OR S9 OR S10 OR S11 OR S12 OR S13 OR S14 | 11 |
| **S16** | **(**MH "Stroke+") OR (MH "Stroke Patients") OR (MH "Stroke Units") OR (MH "Ischemic Stroke+") OR "stroke or cerebrovascular accident or cva" | 6 |
| **S17** | acute stroke or cerebrovascular accident or cva | 4 |
| **S18** | ( cerebrovascular disorders or brain ischemia or stroke ) and ( symptoms or signs or characteristics ) and ( unusual or uncommon ) | 11 |
| **S19** | cerebrovascular disorders or brain ischemia or stroke | 11 |
| **S20** | S16 OR S17 OR S18 OR S19 | 1 |
| **S21** | patient care team or patient care teams or multiprofessional team or patient care | 2,514 |
| **S22** | stroke team | 11 |
| **S23** | (MH "Emergency Service+") OR (MH "Emergency Nursing+") OR (MH "Physicians, Emergency") OR (MH "Emergencies") OR (MH "Emergency Doctor") | 1,536 |
| **S24** | (MH "Radiologists") | 0 |
| **S25** | paramedics or paramedic or emergency medical service or emt or ambulance | 103 |
| **S26** | (MH "Physicians+") OR (MH "Physician Assistants") OR (MH "Physicians, Emergency") | 500 |
| **S27** | nurses or nursing staff or nurse | 52 |
| **S28** | S21 OR S22 OR S23 OR S24 OR S25 OR S26 OR S27 | 63 |
| **S29** | S7 AND S15 AND S20 AND S28 | 0 |

**Database:** EBSCO **(CINAHIL)** Wednesday, May 17, 2023, 3:23:30 PM

| **#** | **Query** | **Results** |
| --- | --- | --- |
| S1 | (MH "Simulations+") OR (MH "Computer Simulation+") OR (MH "Computerized Clinical Simulation Testing") OR (MH "Patient Simulation") OR "simulation training or simulation education or simulation learning" | 54,918 |
| S2 | manikin simulation or manikins or manneqins or human anatomical models or simulation | 1,477 |
| S3 | high fidelity simulation training | 242 |
| S4 | virtual technology simulation | 64 |
| S5 | high fidelity workshop or bootcamp or course | 0 |
| S6 | high fidelity workshop or bootcamp or course | 25 |
| S7 | S1 OR S2 OR S3 OR S4 OR S5 OR S6 | 56,860 |
| S8 | thrombolytic treatment or thombolysis or tissue plasminogen activator or tpa or alteplase | 0 |
| S9 | thrombolytic treatment or thombolysis or tissue plasminogen activator or tpa or alteplase | 4 |
| S10 | (MH "Thrombolytic Therapy") OR "thrombolysis or thrombolytic therapy or fibrinolysis or fibrinolytic therapy" | 8,316 |
| S11 | plasminogen activators or tpa or t-pa or rtpa or rt-pa | 310 |
| S12 | alteplase or tenectaplase | 0 |
| S13 | door to needle time or door to needle time stroke or dtn or door to thrombolysis | 92 |
| S14 | onset to treatment or time to treatment | 376 |
| S15 | S8 OR S9 OR S10 OR S11 OR S12 OR S13 OR S14 | 8,777 |
| S16 | (MH "Stroke+") OR (MH "Stroke Patients") OR (MH "Stroke Units") OR (MH "Ischemic Stroke+") OR "stroke or cerebrovascular accident or cva" | 82,025 |
| S17 | acute stroke or cerebrovascular accident or cva | 1 |
| S18 | ( cerebrovascular disorders or brain ischemia or stroke ) and ( symptoms or signs or characteristics ) and ( unusual or uncommon ) | 0 |
| S19 | cerebrovascular disorders or brain ischemia or stroke | 19,424 |
| S20 | S16 OR S17 OR S18 OR S19 | 82,037 |
| S21 | patient care team or patient care teams or multiprofessional team or patient care | 17 |
| S22 | stroke team | 616 |
| S23 | (MH "Emergency Service+") OR (MH "Emergency Nursing+") OR (MH "Physicians, Emergency") OR (MH "Emergencies") OR (MH "Emergency Patients") | 97,976 |
| S24 | (MH "Radiologists") | 3,930 |
| S25 | paramedics or paramedic or emergency medical service or emt or ambulance | 6 |
| S26 | (MH "Physicians+") OR (MH "Physician Assistants") OR (MH "Physicians, Emergency") | 133,019 |
| S27 | nurses or nursing staff or nurse | 2,595 |
| S28 | S21 OR S22 OR S23 OR S24 OR S25 OR S26 OR S27 | 226202 |
| S29 | S7 AND S15 AND S20 AND S28 | 3 |

**Database:** Elsevier **(Scopus)** Wednesday, May 17, 2023, 21:20 PM

| **#** | **Query** | **Results** |
| --- | --- | --- |
| **1** | TITLE-ABS-KEY ( stroke ) | 518,682 |
| **2** | TITLE-ABS-KEY ( "Ischemic Stroke" ) | 86,774 |
| **3** | TITLE-ABS-KEY ( "Brain Ischemia" ) | 136,168 |
| **4** | TITLE-ABS-KEY ( "Middle Cerebral Artery Infarction" ) | 731 |
| **5** | TITLE-ABS-KEY ( "Cerebrovascular Event" ) | 8,415 |
| **6** | ( TITLE-ABS-KEY ( stroke ) )  OR  ( TITLE-ABS-KEY ( "Ischemic Stroke" ) )  OR  ( TITLE-ABS-KEY ( "Brain Ischemia" ) )  OR  ( TITLE-ABS-KEY ( "Middle Cerebral Artery Infarction" ) )  OR  ( TITLE-ABS-KEY ( "Cerebrovascular Event" ) ) | 581,741 |
| **7** | TITLE-ABS-KEY ( "Tissue Plasminogen Activator" ) | 38,679 |
| **8** | TITLE-ABS-KEY ( tpa ) | 37,258 |
| **9** | TITLE-ABS-KEY ( rtpa ) | 1,698 |
| **10** | TITLE-ABS-KEY ( alteplase ) | 19,612 |
| **11** | TITLE-ABS-KEY ( thrombolysis ) | 38,122 |
| **12** | TITLE-ABS-KEY ( door  AND to  AND needle  OR  dtn  OR  door  AND to  AND treatment ) | 9,742 |
| **13** | TITLE-ABS-KEY ( time  AND to  AND treatment ) | 2,074,476 |
| **14** | ( TITLE-ABS-KEY ( "Tissue Plasminogen Activator" ) )  OR  ( TITLE-ABS-KEY ( tpa ) )  OR  ( TITLE-ABS-KEY ( rtpa ) )  OR  ( TITLE-ABS-KEY ( alteplase ) )  OR  ( TITLE-ABS-KEY ( thrombolysis ) )  OR  ( TITLE-ABS-KEY ( door  AND to  AND needle  OR  dtn  OR  door  AND to  AND treatment ) )  OR  ( TITLE-ABS-KEY ( time  AND to  AND treatment ) ) | 2,168,153 |
| **15** | TITLE-ABS-KEY ( health  AND care  AND professional  OR  healthcare  AND team ) | 45,711 |
| **16** | TITLE-ABS-KEY ( multidisciplinary  AND team  OR  mdt ) | 63,041 |
| **17** | TITLE-ABS-KEY ( doctor  OR  physician  OR  emergency  AND doctor ) | 333,123 |
| **18** | TITLE-ABS-KEY ( stroke  AND team  OR  critical  AND care  OR  accident  AND  emergency ) | 1,851 |
| **19** | TITLE-ABS-KEY ( nurse  OR  nurse  AND team ) | 40,123 |
| **20** | TITLE-ABS-KEY ( radiologist  OR  stroke  AND doctor ) | 4,616 |
| **21** | ( TITLE-ABS KEY ( health  AND care  AND professional  OR  healthcare  AND team ) )  OR  #  TITLE-ABS-KEY ( multidisciplinary AND team OR mdt )  OR  ( TITLE-ABS-KEY ( doctor  OR  physician  OR  emergency  AND doctor ) )  OR  ( TITLE-ABS-KEY ( stroke  AND team  OR  critical  AND care  OR  accident  AND  emergency ) )  OR  ( TITLE-ABS-KEY ( nurse  OR  nurse  AND team ) )  OR  ( TITLE-ABS-KEY ( radiologist  OR  stroke  AND doctor ) ) | 17,119 |
| **22** | TITLE-ABS-KEY ( simulation  AND training  OR  simulated-based  AND training ) | 104,051 |
| **23** | TITLE-ABS-KEY ( simulat*  W/5  ( educat*  OR  cours*  OR  workshop*  OR  bootcamp*  OR  boot-camp*  OR  learn*  OR  experienc*  OR  curricul*  OR  framework*  OR  teach*  OR  guid*  OR  coach*  OR  supervis*  OR  seminar*  OR  lectur*  OR  round*  OR  tutor*  OR  mentor*  OR  program*  OR  traning*  OR  event* ) ) | 241,780 |
| **24** | TITLE-ABS-KEY ( high  W/3  fidelity*  W/5  ( educat*  OR  cours*  OR  workshop*  OR  bootcamp*  OR  boot-camp*  OR  learn*  OR  experienc*  OR  curricul*  OR  framework*  OR  teach*  OR  guid*  OR  coach*  OR  supervis*  OR  seminar*  OR  lectur*  OR  round*  OR  tutor*  OR  mentor*  OR  program*  OR  traning*  OR  event* ) ) | 3,399 |
| **25** | TITLE-ABS-KEY ( hi-fi*  W/5  ( educat*  OR  cours*  OR  workshop*  OR  bootcamp*  OR  boot-camp*  OR  learn*  OR  experienc*  OR  curricul*  OR  framework*  OR  teach*  OR  guid*  OR  coach*  OR  supervis*  OR  seminar*  OR  lectur*  OR  round*  OR  tutor*  OR  mentor*  OR  program*  OR  traning*  OR  event* ) ) | 38 |
| **26** | TITLE-ABS-KEY ( hi-fi*  W/5  ( educat*  OR  cours*  OR  workshop*  OR  bootcamp*  OR  boot-camp*  OR  learn*  OR  experienc*  OR  curricul*  OR  framework*  OR  teach*  OR  guid*  OR  coach*  OR  supervis*  OR  seminar*  OR  lectur*  OR  round*  OR  tutor*  OR  mentor*  OR  program*  OR  traning*  OR  event* ) ) | 38 |
| **27** | TITLE-ABS-KEY ( in  W/3  situ*  W/5  ( educat*  OR  cours*  OR  workshop*  OR  bootcamp*  OR  boot-camp*  OR  learn*  OR  experienc*  OR  curricul*  OR  framework*  OR  teach*  OR  guid*  OR  coach*  OR  supervis*  OR  seminar*  OR  lectur*  OR  round*  OR  tutor*  OR  mentor*  OR  program*  OR  traning*  OR  event* ) ) | 44,441 |
| **28** | TITLE-ABS-KEY ( insitu*  W/5  ( educat*  OR  cours*  OR  workshop*  OR  bootcamp*  OR  boot-camp*  OR  learn*  OR  experienc*  OR  curricul*  OR  framework*  OR  teach*  OR  guid*  OR  coach*  OR  supervis*  OR  seminar*  OR  lectur*  OR  round*  OR  tutor*  OR  mentor*  OR  program*  OR  traning*  OR  event* ) ) | 102 |
| **29** | TITLE-ABS-KEY ( simulation*  W/5  ( educat*  OR  cours*  OR  workshop*  OR  bootcamp*  OR  boot-camp*  OR  learn*  OR  experienc*  OR  curricul*  OR  framework*  OR  teach*  OR  guid*  OR  coach*  OR  supervis*  OR  seminar*  OR  lectur*  OR  round*  OR  tutor*  OR  mentor*  OR  program*  OR  traning*  OR  event* ) ) | 182,625 |
| **30** | TITLE-ABS-KEY ( full  W/3  scale*  W/5  ( educat*  OR  cours*  OR  workshop*  OR  bootcamp*  OR  boot-camp*  OR  learn*  OR  experienc*  OR  curricul*  OR  framework*  OR  teach*  OR  guid*  OR  coach*  OR  supervis*  OR  seminar*  OR  lectur*  OR  round*  OR  tutor*  OR  mentor*  OR  program*  OR  traning*  OR  event* ) ) | 4,124 |
| **31** | ( TITLE-ABS-KEY ( simulation  AND training  OR  simulated-based  AND training ) )  OR  ( TITLE-ABS-KEY ( simulat*  W/5  ( educat*  OR  cours*  OR  workshop*  OR  bootcamp*  OR  boot-camp*  OR  learn*  OR  experienc*  OR  curricul*  OR  framework*  OR  teach*  OR  guid*  OR  coach*  OR  supervis*  OR  seminar*  OR  lectur*  OR  round*  OR  tutor*  OR  mentor*  OR  program*  OR  traning*  OR  event* ) ) )  OR  ( TITLE-ABS-KEY ( high  W/3  fidelity*  W/5  ( educat*  OR  cours*  OR  workshop*  OR  bootcamp*  OR  boot-camp*  OR  learn*  OR  experienc*  OR  curricul*  OR  framework*  OR  teach*  OR  guid*  OR  coach*  OR  supervis*  OR  seminar*  OR  lectur*  OR  round*  OR  tutor*  OR  mentor*  OR  program*  OR  traning*  OR  event* ) ) )  OR  ( TITLE-ABS-KEY ( hi-fi*  W/5  ( educat*  OR  cours*  OR  workshop*  OR  bootcamp*  OR  boot-camp*  OR  learn*  OR  experienc*  OR  curricul*  OR  framework*  OR  teach*  OR  guid*  OR  coach*  OR  supervis*  OR  seminar*  OR  lectur*  OR  round*  OR  tutor*  OR  mentor*  OR  program*  OR  traning*  OR  event* ) ) )  OR  ( TITLE-ABS-KEY ( hi-fi*  W/5  ( educat*  OR  cours*  OR  workshop*  OR  bootcamp*  OR  boot-camp*  OR  learn*  OR  experienc*  OR  curricul*  OR  framework*  OR  teach*  OR  guid*  OR  coach*  OR  supervis*  OR  seminar*  OR  lectur*  OR  round*  OR  tutor*  OR  mentor*  OR  program*  OR  traning*  OR  event* ) ) )  OR  ( TITLE-ABS-KEY ( in  W/3  situ*  W/5  ( educat*  OR  cours*  OR  workshop*  OR  bootcamp*  OR  boot-camp*  OR  learn*  OR  experienc*  OR  curricul*  OR  framework*  OR  teach*  OR  guid*  OR  coach*  OR  supervis*  OR  seminar*  OR  lectur*  OR  round*  OR  tutor*  OR  mentor*  OR  program*  OR  traning*  OR  event* ) ) )  OR  ( TITLE-ABS-KEY ( insitu*  W/5  ( educat*  OR  cours*  OR  workshop*  OR  bootcamp*  OR  boot-camp*  OR  learn*  OR  experienc*  OR  curricul*  OR  framework*  OR  teach*  OR  guid*  OR  coach*  OR  supervis*  OR  seminar*  OR  lectur*  OR  round*  OR  tutor*  OR  mentor*  OR  program*  OR  traning*  OR  event* ) ) )  OR  ( TITLE-ABS-KEY ( simulation*  W/5  ( educat*  OR  cours*  OR  workshop*  OR  bootcamp*  OR  boot-camp*  OR  learn*  OR  experienc*  OR  curricul*  OR  framework*  OR  teach*  OR  guid*  OR  coach*  OR  supervis*  OR  seminar*  OR  lectur*  OR  round*  OR  tutor*  OR  mentor*  OR  program*  OR  traning*  OR  event* ) ) )  OR  ( TITLE-ABS-KEY ( full  W/3  scale*  W/5  ( educat*  OR  cours*  OR  workshop*  OR  bootcamp*  OR  boot-camp*  OR  learn*  OR  experienc*  OR  curricul*  OR  framework*  OR  teach*  OR  guid*  OR  coach*  OR  supervis*  OR  seminar*  OR  lectur*  OR  round*  OR  tutor*  OR  mentor*  OR  program*  OR  traning*  OR  event* ) ) ) | 373,742 |
| **32** | ( ( TITLE-ABS-KEY ( stroke ) )  OR  ( TITLE-ABS-KEY ( "Ischemic Stroke" ) )  OR  ( TITLE-ABS-KEY ( "Brain Ischemia" ) )  OR  ( TITLE-ABS-KEY ( "Middle Cerebral Artery Infarction" ) )  OR  ( TITLE-ABS-KEY ( "Cerebrovascular Event" ) ) )  AND  ( ( TITLE-ABS-KEY ( "Tissue Plasminogen Activator" ) )  OR  ( TITLE-ABS-KEY ( tpa ) )  OR  ( TITLE-ABS-KEY ( rtpa ) )  OR  ( TITLE-ABS-KEY ( alteplase ) )  OR  ( TITLE-ABS-KEY ( thrombolysis ) )  OR  ( TITLE-ABS-KEY ( door  AND to  AND needle  OR  dtn  OR  door  AND to  AND treatment ) )  OR  ( TITLE-ABS-KEY ( time  AND to  AND treatment ) ) )  AND  ( ( TITLE-ABS-KEY ( health  AND care  AND professional  OR  healthcare  AND team ) )  OR  #  31  OR  ( TITLE-ABS-KEY ( doctor  OR  physician  OR  emergency  AND doctor ) )  OR  ( TITLE-ABS-KEY ( stroke  AND team  OR  critical  AND care  OR  accident  AND  emergency ) )  OR  ( TITLE-ABS-KEY ( nurse  OR  nurse  AND team ) )  OR  ( TITLE-ABS-KEY ( radiologist  OR  stroke  AND doctor ) ) )  AND  ( ( TITLE-ABS-KEY ( simulation  AND training  OR  simulated-based  AND training ) )  OR  ( TITLE-ABS-KEY ( simulat*  W/5  ( educat*  OR  cours*  OR  workshop*  OR  bootcamp*  OR  boot-camp*  OR  learn*  OR  experienc*  OR  curricul*  OR  framework*  OR  teach*  OR  guid*  OR  coach*  OR  supervis*  OR  seminar*  OR  lectur*  OR  round*  OR  tutor*  OR  mentor*  OR  program*  OR  traning*  OR  event* ) ) )  OR  ( TITLE-ABS-KEY ( high  W/3  fidelity*  W/5  ( educat*  OR  cours*  OR  workshop*  OR  bootcamp*  OR  boot-camp*  OR  learn*  OR  experienc*  OR  curricul*  OR  framework*  OR  teach*  OR  guid*  OR  coach*  OR  supervis*  OR  seminar*  OR  lectur*  OR  round*  OR  tutor*  OR  mentor*  OR  program*  OR  traning*  OR  event* ) ) )  OR  ( TITLE-ABS-KEY ( hi-fi*  W/5  ( educat*  OR  cours*  OR  workshop*  OR  bootcamp*  OR  boot-camp*  OR  learn*  OR  experienc*  OR  curricul*  OR  framework*  OR  teach*  OR  guid*  OR  coach*  OR  supervis*  OR  seminar*  OR  lectur*  OR  round*  OR  tutor*  OR  mentor*  OR  program*  OR  traning*  OR  event* ) ) )  OR  ( TITLE-ABS-KEY ( hi-fi*  W/5  ( educat*  OR  cours*  OR  workshop*  OR  bootcamp*  OR  boot-camp*  OR  learn*  OR  experienc*  OR  curricul*  OR  framework*  OR  teach*  OR  guid*  OR  coach*  OR  supervis*  OR  seminar*  OR  lectur*  OR  round*  OR  tutor*  OR  mentor*  OR  program*  OR  traning*  OR  event* ) ) )  OR  ( TITLE-ABS-KEY ( in  W/3  situ*  W/5  ( educat*  OR  cours*  OR  workshop*  OR  bootcamp*  OR  boot-camp*  OR  learn*  OR  experienc*  OR  curricul*  OR  framework*  OR  teach*  OR  guid*  OR  coach*  OR  supervis*  OR  seminar*  OR  lectur*  OR  round*  OR  tutor*  OR  mentor*  OR  program*  OR  traning*  OR  event* ) ) )  OR  ( TITLE-ABS-KEY ( insitu*  W/5  ( educat*  OR  cours*  OR  workshop*  OR  bootcamp*  OR  boot-camp*  OR  learn*  OR  experienc*  OR  curricul*  OR  framework*  OR  teach*  OR  guid*  OR  coach*  OR  supervis*  OR  seminar*  OR  lectur*  OR  round*  OR  tutor*  OR  mentor*  OR  program*  OR  traning*  OR  event* ) ) )  OR  ( TITLE-ABS-KEY ( simulation*  W/5  ( educat*  OR  cours*  OR  workshop*  OR  bootcamp*  OR  boot-camp*  OR  learn*  OR  experienc*  OR  curricul*  OR  framework*  OR  teach*  OR  guid*  OR  coach*  OR  supervis*  OR  seminar*  OR  lectur*  OR  round*  OR  tutor*  OR  mentor*  OR  program*  OR  traning*  OR  event* ) ) )  OR  ( TITLE-ABS-KEY ( full  W/3  scale*  W/5  ( educat*  OR  cours*  OR  workshop*  OR  bootcamp*  OR  boot-camp*  OR  learn*  OR  experienc*  OR  curricul*  OR  framework*  OR  teach*  OR  guid*  OR  coach*  OR  supervis*  OR  seminar*  OR  lectur*  OR  round*  OR  tutor*  OR  mentor*  OR  program*  OR  traning*  OR  event* ) ) ) ) | 2 |

Conference abstracts were identified from EMBASE, PubMed, Scopus and Google Scholar database searches. The search strategy for the conference databases included terms for the participants such as: ‘healthcare team/professionals’, ‘patient care team’, ‘stroke team’, and ‘multidisciplinary team’. Terms used for intervention included: ‘simulation-based training’, ‘high/low fidelity simulation training’, ‘manikin, ‘in-situ education’ and ‘simulation workshops’. Topics included ‘ischaemic stroke’, ‘acute stroke’, and ‘cerebrovascular disorders’. Terms for outcomes included ‘thrombolysis’, ‘fibrinolysis’, ‘tissue plasminogen activator’, ‘door-to-needle’ and ‘door-to-treatment’.

**APPENDIX 3 – Sample Data Extraction Sheet**

| **Basic Characteristics** | **Participates and Setting** | **Intervention (Simulation Training)** | **Outcomes** |
| --- | --- | --- | --- |
| 1. Author: 2. Year: 3. Title of Paper: 4. Country: 5. Type of study: 6. When did the study take place? | 1. Who were the participants? 2. How were the pre-intervention study participants identified? 3. How many participants (number of learners) pre- and post-intervention or how many participants in each session? 4. How were the participants recruited? 5. Who were the simulated participants? 6. How many patients were in the pre-intervention group? 7. How many patients were in the intervention group? 8. Where was the study conducted? 9. Which hospital setting did the study take place in? | 1. What was the type and of simulation sessions (definition)? 2. What was the number of simulation sessions? 3. What was the length of the simulation sessions? 4. What did they use for simulation? 5. What is reported in the way of debriefing? | 1. How were the door-to-needle times recorded pre-simulation? 2. How were the door-to-needle times recorded post-simulation? 3. What was the door-to-needle time pre-intervention? 4. What was the intervention group door-to-needle time? 5. p-value? 6. Any confounders? 7. Limitations of study? 8. Any secondary outcomes (common themes within studies)? |

**APPENDIX 4 – Details on the Assessment of Risk of Bias ROBINS-I Results**

| **Study** | Bias due to confounding | Bias in selection of participants into the study | Bias in classification of interventions | Bias due to deviations from intended interventions | Bias due to missing data | Bias in measurement of outcomes | Bias in selection of the reported result | Overall certainty of evidence |
| --- | --- | --- | --- | --- | --- | --- | --- | --- |
| **Tahtali et al., 2016** | No information on confounders or adjustment for confounders | Low risk of bias (all eligible participants included in study; start of follow-up and intervention coincide) | Moderate risk of bias (some intervention status determined retrospectively) | Moderate risk of bias (deviations from intended intervention with slight impact) | No information about missing data or potential for missing data | Low risk of bias (outcome assessment comparable across groups) | Moderate risk of bias (no indication of selection of reported analysis) | Moderate  (sound evidence but not like a well-performed randomised trial) |
| **Waterson et al., 2016** | No information on confounders or adjustment for confounders | Low risk of bias (all eligible participants included in study; start of follow-up and intervention coincide) | Low risk of bias (intervention well-defined and definition based on information at time of intervention) | Low risk of bias (deviations unlikely to impact outcome) | No information about missing data or potential for missing data | Low risk of bias (outcome assessment comparable across groups) | No information available | No information |
| **Ohara et al., 2017** | No information on confounders or adjustment for confounders | Low risk of bias (all eligible participants included in study; start of follow-up and intervention coincide) | Low risk of bias  (intervention well-defined and definition based on information at time of intervention) | Low risk of bias (deviations unlikely to impact outcome) | Low risk of bias (data complete) | Low risk of bias (outcome assessment comparable across groups) | Moderate risk of bias (no indication of selection of reported analysis) | Moderate  (sound evidence but not like a well-performed randomised trial) |
| **Richardson et al., 2017** | No information on confounders or adjustment for confounders | Low risk of bias (all eligible participants included in study; start of follow-up and intervention coincide) | Low risk of bias  (intervention well-defined and definition based on information at time of intervention) | Low risk of bias (deviations unlikely to impact outcome) | No information about missing data or potential for missing data | Low risk of bias (outcome assessment comparable across groups) | Moderate risk of bias (no indication of selection of reported analysis) | No information |
| **Ruff et al., 2017** | No information on confounders or adjustment for confounders | Low risk of bias (all eligible participants included in study; start of follow-up and intervention coincide) | Low risk of bias  (intervention well-defined and definition based on information at time of intervention) | Low risk of bias (deviations unlikely to impact outcome) | Moderate (analysis unlikely to have removed risk of bias from missing data) | Low risk of bias (outcome assessment comparable across groups) | Moderate risk of bias (no indication of selection of reported analysis) | Moderate  (sound evidence but not like a well-performed randomised trial) |
| **Tahtali et al., 2017** | Low risk of bias confounders adjusted | Low risk of bias (all eligible participants included in study; start of follow-up and intervention coincide) | Moderate risk of bias (some aspects of assignment of intervention determined retrospectively) | Low risk of bias (deviations unlikely to impact outcome) | No information about missing data or potential for missing data | Moderate risk of bias (minimal influence of knowledge of participants on outcome measures) | Moderate risk of bias (no indication of selection of reported analysis) | Moderate  (sound evidence but not like a well-performed randomised trial) |
| **Tse-Chang et al., 2017** | No information on confounders or adjustment for confounders | Low risk of bias (all eligible participants included in study; start of follow-up and intervention coincide) | Low risk of bias  (intervention well-defined and definition based on information at time of intervention) | Low risk of bias (deviations unlikely to impact outcome) | No information about missing data or potential for missing data | No information on methods of outcome assessment | No information available | No information |
| **Windle et al., 2017** | Moderate risk of bias appropriate adjustment for confounders | Low risk of bias (all eligible participants included in study; start of follow-up and intervention coincide) | Low risk of bias  (intervention well-defined and definition based on information at time of intervention) | Low risk of bias (deviations unlikely to impact outcome) | No information about missing data or potential for missing data | Low risk of bias (outcome assessment comparable across groups) | No information available | No information |
| **Zidan et al., 2017** | Moderate risk of bias appropriate adjustment for confounders | Low risk of bias (all eligible participants included in study; start of follow-up and intervention coincide) | Moderate risk of bias (some aspects of assignment of intervention determined retrospectively) | Low risk of bias (deviations unlikely to impact outcome) | No information about missing data or potential for missing data | Low risk of bias (outcome assessment comparable across groups) | Low risk of bias (all reported results correspond to intended outcomes and analyses) | Moderate  (sound evidence but not like a well-performed randomised trial) |
| **Carvalho et al., 2018** | Low risk of bias confounders adjusted | Moderate risk of bias (selection of participants may have been related to intervention and outcome; appropriate methods used to adjust for selection bias) | Moderate risk of bias (some aspects of assignment of intervention determined retrospectively) | Low risk of bias (deviations unlikely to impact outcome) | No information about missing data or potential for missing data | Low risk of bias (outcome assessment comparable across groups) | Moderate risk of bias (no indication of selection of reported analysis) | Moderate  (sound evidence but not like a well-performed randomised trial) |
| **Haesebaert et al., 2018** | Low risk of bias confounders adjusted | Low risk of bias (all eligible participants included in study; start of follow-up and intervention coincide) | Low risk of bias  (intervention well-defined and definition based on information at time of intervention) | Low risk of bias (deviations unlikely to impact outcome) | Low risk of bias (data complete) | Low risk of bias (outcome assessment comparable across groups) | Low risk of bias (all reported results correspond to intended outcomes and analyses) | Low (comparable to a well-performed randomised trial) |
| **Mehta et al., 2018** | Moderate risk of bias appropriate adjustment for confounders | Low risk of bias (all eligible participants included in study; start of follow-up and intervention coincide) | Low risk of bias  (intervention well-defined and definition based on information at time of intervention) | Moderate risk of bias (deviations from intended intervention with slight impact) | No information about missing data or potential for missing data | Low risk of bias (outcome assessment comparable across groups) | Moderate risk of bias (no indication of selection of reported analysis) | Moderate  (sound evidence but not like a well-performed randomised trial) |
| **Sanders et al, 2018** | No information on confounders or adjustment for confounders | Low risk of bias (all eligible participants included in study; start of follow-up and intervention coincide) | Low risk of bias  (intervention well-defined and definition based on information at time of intervention) | No information reported | No information about missing data or potential for missing data | Low risk of bias (outcome assessment comparable across groups) | No information available | No information |
| **Ajmi et al., 2019** | Serious risk of bias due to insufficient adjustment for confounders | Low risk of bias (all eligible participants included in study; start of follow-up and intervention coincide) | Moderate risk of bias (some aspects of assignment of intervention determined retrospectively) | Low risk of bias (deviations unlikely to impact outcome) | Serious (missing data inappropriately addressed in analysis and proportions of participants differed across interventions) | Moderate risk of bias (minimal influence of knowledge of participants on outcome measures) | Moderate risk of bias (no indication of selection of reported analysis) | Serious (problems with addressing missing data and adjusting for confounders) |
| **Singh et al., 2019** | No information on confounders or adjustment for confounders | Low risk of bias (all eligible participants included in study; start of follow-up and intervention coincide) | Low risk of bias  (intervention well-defined and definition based on information at time of intervention) | Low risk of bias (deviations unlikely to impact outcome) | No information about missing data or potential for missing data | Low risk of bias (outcome assessment comparable across groups) | No information available | No information |
| **Bubel et al., 2020** | Moderate risk of bias appropriate adjustment for confounders | Low risk of bias (all eligible participants included in study; start of follow-up and intervention coincide) | Low risk of bias  (intervention well-defined and definition based on information at time of intervention) | Low risk of bias (deviations unlikely to impact outcome) | No information about missing data or potential for missing data | Low risk of bias (outcome assessment comparable across groups) | No information available | No information |
| **Bohmann et al., 2022** | Moderate risk of bias appropriate adjustment for confounders | Low risk of bias (all eligible participants included in study; start of follow-up and intervention coincide) | Low risk of bias  (intervention well-defined and definition based on information at time of intervention) | Low risk of bias (deviations unlikely to impact outcome) | Moderate (analysis unlikely to have removed risk of bias from missing data) | Low risk of bias (outcome assessment comparable across groups) | Moderate risk of bias (no indication of selection of reported analysis) | Moderate  (sound evidence but not like a well-performed randomised trial) |
| **Rhew et al., 2022** | No information on confounders or adjustment for confounders | Low risk of bias (all eligible participants included in study; start of follow-up and intervention coincide) | Low risk of bias  (intervention well-defined and definition based on information at time of intervention) | Low risk of bias (deviations unlikely to impact outcome) | No information about missing data or potential for missing data | Low risk of bias (outcome assessment comparable across groups) | No information available | No information |
| **Svobodova et al., 2023** | No information on confounders or adjustment for confounders | Low risk of bias (all eligible participants included in study; start of follow-up and intervention coincide) | Moderate risk of bias (some aspects of assignment of intervention determined retrospectively) | Low risk of bias (deviations unlikely to impact outcome) | No information about missing data or potential for missing data | Low risk of bias (outcome assessment comparable across groups) | Serious risk of bias (high risk for selective reporting from multiple analyses) | Serious (problem with reporting of results from multiple analyses) |

**APPENDIX 5 – Table of Excluded Studies**

| **Author, Year** | **Reason for Exclusion** | **Author, Year** | **Reason for Exclusion** |
| --- | --- | --- | --- |
| Stahl et al., 2003 | Review | McKee et al., 2020 | Book |
| Misra et al., 2005 | Review | Saver, 2006 | Commentary |
| Johansson et al., 2010 | Review | Murray, 2015 | Commentary |
| Desai et al., 2013 | Review | Lahr et al., 2016 | Commentary |
| Kamal et al., 2018 | Review | Josephson and Hooman, 2018 | Commentary |
| Moreno et al., 2019 | Review | Li et al., 2020 | Commentary |
| Hasnain et al., 2020 | Review | Lockey et al., 2017 | Editorial |
| Mass et al., 2020 | Review | Brazil et al., 2019 | Editorial |
| Siarkowski et al., 2020 | Review | Lahr et al., 2012 | Letter |
| Zhu et al., 2020 | Review | Gutierrez et al., 2016 | Letter |
| Allen et al., 2022 | Review | Miarons, 2017 | Letter |
| Patil et al., 2022 | Review | Vecchiato, 2017 | Letter |
| Nguyen et al., 2023 | Review | Kvernland et al., 2019 | Letter |
| Price et al., 2023 | Review | Sigounas, 2019 | News and Opinion |
| Binning et al., 2013 | Book | Klingner, 2015 | Short Case Report |
| Watkins et al., 2019 | Book | Brown and Elofuke, 2021 | Short Report |
